# Terahertz emission from diamond nitrogen-vacancy centers

**DOI:** 10.1126/sciadv.adn0616

**Published:** 2024-05-29

**Authors:** Sándor Kollarics, Bence Gábor Márkus, Robin Kucsera, Gergő Thiering, Ádám Gali, Gergely Németh, Katalin Kamarás, László Forró, Ferenc Simon

**Affiliations:** ^1^ Department of Physics, Institute of Physics, Budapest University of Technology and Economics, Műegyetem rkp.3, H-1111 Budapest, Hungary.; ^2^ ELKH-BME Condensed Matter Research Group, Budapest University of Technology and Economics, Műegyetem rkp. 3, H-1111 Budapest, Hungary.; ^3^ Institute for Solid State Physics and Optics, HUN-REN Wigner Research Centre for Physics, PO. Box 49, H-1525 Budapest, Hungary.; ^4^ Stavropoulos Center for Complex Quantum Matter, Department of Physics and Astronomy, University of Notre Dame, Notre Dame, IN 46556, USA.; ^5^ Department of Atomic Physics, Institute of Physics, Budapest University of Technology and Economics, Műegyetem rkp. 3, H-1111 Budapest, Hungary.; ^6^ MTA-WFK “Lendület” Momentum Semiconductor Nanostructures Research Group, PO. Box 49, H-1525 Budapest, Hungary.; ^7^ Institute of Technical Physics and Materials Science, HUN-REN Centre for Energy Research, P.O. Box 49, H-1525 Budapest, Hungary.; ^8^ Laboratory of Physics of Complex Matter, École Polytechnique Fédérale de Lausanne, Lausanne CH-1015, Switzerland.

## Abstract

Coherent light sources emitting in the terahertz range are highly sought after for fundamental research and applications. Terahertz lasers rely on achieving population inversion. We demonstrate the generation of terahertz radiation using nitrogen-vacancy centers in a diamond single crystal. Population inversion is achieved through the Zeeman splitting of the *S* = 1 state in 15 tesla, resulting in a splitting of 0.42 terahertz, where the middle *S_z _*= 0 sublevel is selectively pumped by visible light. To detect the terahertz radiation, we use a phase-sensitive terahertz setup, optimized for electron spin resonance (ESR) measurements. We determine the spin-lattice relaxation time up to 15 tesla using the light-induced ESR measurement, which shows the dominance of phonon-mediated relaxation and the high efficacy of the population inversion. The terahertz radiation is tunable by the magnetic field, thus these findings may lead to the next generation of tunable coherent terahertz sources.

## Teaser

Optically induced spin polarization of nitrogen-vacancy centers in high magnetic fields could be a source of terahertz radiation.

## INTRODUCTION

Despite considerable efforts, the domain known as the terahertz gap (0.1 to 10 THz) remains one of the less explored and exploited ranges of the electromagnetic spectrum. This is mainly due to a lack of coherent radiation sources between electronics-based microwave devices (operating range below 0.1 THz) and infrared lasers, operating above 100 cm^–1^ (3 THz). The quest for coherent terahertz sources is motivated by both fundamental research and foreseen applications. Examples of the earlier include the study of correlated matter [superconductors (*1*), density wave systems (*2*), exotic magnets (*3*)], nonequilibrium charge dynamics (*4*), and otherwise metastable states (*5*). For applications, terahertz radiation is expected to revolutionize various areas including broadband communication, medical imaging, fusion research, and security applications (*6*).

Incoherent terahertz sources are black-body radiators such as mercury lamps or light-gated Auston switches (*7*), coherent sources include laser frequency mixing- (*8*) and Josephson effect–based devices (*9*), resonant-tunneling diodes (*10*), electron tubes (*11*), quantum-cascade lasers (*12*), and relativistic-electron sources using synchrotrons (*13*) or free-electron lasers (*14*). Much as these devices satisfy various requirements (including high power, pulsed nature, and tunability), it would be highly desired to present a source that operates based on the same basic laser principle that was originally used in ruby (*15*): a solid-state system, optically pumped to population inversion, where efficient stimulated emission occurs.

Of the known solid-state systems with suitable energy level structure, the nitrogen-vacancy (NV) center in diamond [more precisely its negative charge state NV(−) with *S* = 1 (*16*)] is unique: It can be efficiently pumped with visible light to selectively populate the *S_z_* = 0 level of the ground state triplet while depleting the *S_z_* = ±1 levels (*17*). In addition, the spin-lattice relaxation time is about a few milliseconds at room temperature (*18*–*20*), which is long enough to maintain the light-induced population. Thus, in a sufficiently strong magnetic field, a population inversion occurs between the upper-lying *S_z_* = 0 and the lower-lying *S_z_* = −1 states. In addition, the NV center is known to be extremely photostable: No photobleaching occurs, which is usually the limiting factor for single-molecule emitters, e.g., the rhodamine dyes. These properties were exploited to yield the NV center–based maser (*21*), which operates at 9.2 GHz, and an NV-based amplifier at 18 GHz (*22*). We note that non-diamond-based alternative quantum emitter systems are also actively researched (*23*).

Here, we observe emission at 0.42 THz (and at 0.21 THz) from NV centers in single crystals of diamond subjected to a 15 T (7.5 T) magnetic field. A phase-sensitive terahertz detector attests that the emitted terahertz photons are coherent with the incoming excitation, whose energy matches the Zeeman splitting between the two states with the inverted population. We also study the sensitive dependence of the population inversion efficiency of the NV center axis orientation with respect to the external magnetic field. We show that the spin-selective intersystem crossing (ISC) rates and thus the optical spin-polarization processes are primarily determined by the orbitals within the *C*_3*v*_ crystal field symmetry even at magnetic fields up to 15 T. The spin-lattice relaxation time of the NV centers down to 20 K is measured using a double-modulated electron spin resonance (ESR) experiment. The result excludes the presence of a relaxation time speeding up due to the magnetic field, thus population inversion can be effectively maintained, which makes the NV system a promising candidate for a coherent terahertz source.

## RESULTS

**Fig. 1.**
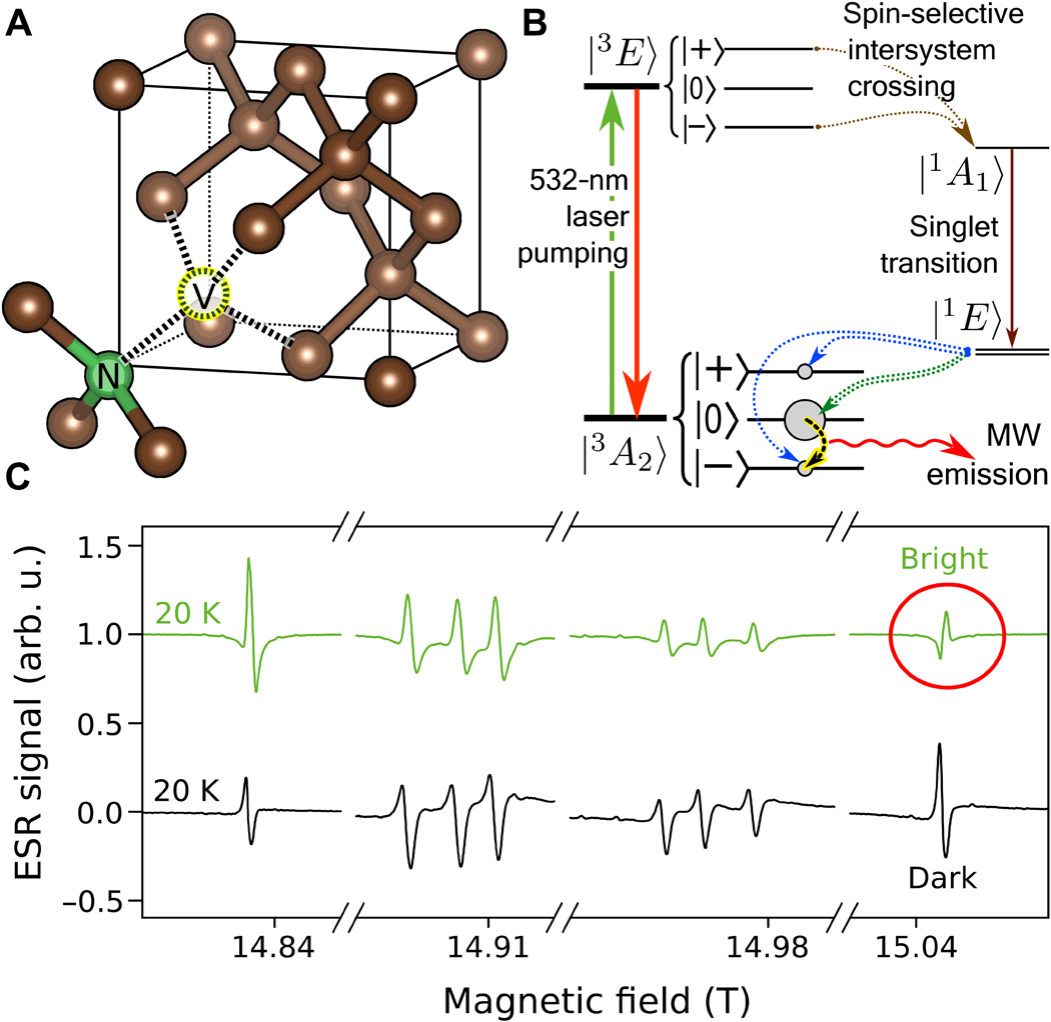
Structure, energy level diagram, and electron spin resonance spectra of NV centers. (**A**) Structure of the nitrogen-vacancy center in diamond. (**B**) Energy level diagram and population of the three sublevels of the *S* = 1 ground state triplet in the dark and under intensive illumination (bright). We show the nonradiative ISC rates from the excited triplet ∣^3^*E*〉 into the metastable singlet state ∣*s*〉 and toward the ground state triplet ∣^3^*A*_2_〉. The ISC from the ∣±〉 sublevels of ∣^3^*E*〉 is much stronger than from the ∣0〉. This, under intensive laser irradiation, leads to the pumping of all electrons to the ∣0〉 state of ∣^3^*A*_2_〉. This is also confirmed by our experiments. In a sufficiently high magnetic field, this corresponds to a population inverted scenario. (**C**) High-field ESR spectra of the NV centers at 20 K in the dark and under laser illumination. The ESR signal of charge-neutral nitrogen atoms (also known as the P1 signal) was removed from the center of the spectrum to improve clarity. Note that the uppermost signal (indicated by a circle) reverses, which is clear evidence of a population inversion. arb. u., arbitrary units.

Nitrogen is a common substitutional center in diamonds. In type Ib diamond, nitrogen atoms are dispersed and they can form the so-called NV center if a vacancy is captured on a neighboring site. Its structure is shown in Fig. 1A. The vacancies are naturally present in the samples or are induced by irradiation and these become mobile at high temperatures, thus efficiently forming the thermally stable NV center (*24*–*26*). The interest is focused on the negatively charged state of the center or NV(−), where the source of the electron is an ionized nitrogen atom (*27*). For the sake of simplicity, we denote the NV(−) centers as NV in the following.

The ground state of the NV center is an *S* = 1 triplet state, where in the absence of an external magnetic field, the *S_z_* = 0 level is separated from the degenerate *S_z_* = ±1 levels by the zero-field splitting (ZFS) parameter of *D*/*h* ≈ 2.87 GHz. These levels are split in a strong magnetic field. The Jablonski diagram in Fig. 1B shows the excited and intermediate levels, as well as the possible transitions. The NV centers absorb light between 450 and 637 nm. Although, in dark conditions, the respective populations of the ground state triplet are given by a Boltzmann distribution, under the intensive irradiation, the *S_z_* = 0 level is selectively populated because of the spin-orbit coupling induced selective ISC (*28*), whereas the *S_z_* = ±1 levels are depleted (*17*, *29*). This causes a population inversion between the *S_z_* = −1 and *S_z_* = 0 levels in magnetic fields above ∼100 mT, which leads to a stimulated microwave emission (or masing effect) when the frequency of the energy splitting lies in the microwave range (*B* ≲ 1 T). This effect opens the possibility for a tunable maser source (*21*, *22*) and for a terahertz maser or terahertz amplifier in a high magnetic field. A rigorous theoretical description can be found in the Supplementary Materials.

Figure 1C shows the high-field/high-frequency ESR spectrum of the NV centers using terahertz radiation of 0.42 THz. The instrument setup and the sample are discussed in Materials and Methods. Briefly, the home-built high-field ESR instrument has a sensitivity of $3\cdot 10^{10}\;\text{spins}/10^{-4}T\sqrt{\text{Hz}}$ and it uses a phase-coherent homodyne detection (*30*, *31*). The sample contains 12 ppm of NV centers, which is one of the highest concentrations reported to date (*32*).

The ESR experiment was performed in the dark and under laser illumination, where a 532 nm laser at 100 mW power and with a beam diameter of about 3 mm excited a sample of a similar area. Conventional ESR (*33*) is based on observing the resonant absorption of coherent radiation between adjacent levels (Δ*m* = 1), which are the eigenstates of the Zeeman Hamiltonian. In a typical ESR experiment, the microwave irradiation frequency is kept constant and the magnetic field is swept as shown in Fig. 1C. Note that the eight observed ESR transitions look to be similar for the dark spectrum, i.e., they show an identical phase.

The spin Hamiltonian of the NV center reads

$$\hat{H}=D\left[S_{z}^{2}-\frac{1}{3}S\left(S+1\right)\right]+g_{e}\mu_{B}\text{BS}$$ (1)

This Hamiltonian is given in a coordinate system whose *z* axis is fixed to the NV axis. The first term is the zero-field Hamiltonian with *D*/*h* ≈ 2.87 GHz. The second term describes the Zeeman effect with the *g* factor, *g_e_* ≈ 2.0029 (*34*) and **B** can have an arbitrary direction with respect to the NV axis. The NV axis can take four inequivalent directions for an arbitrary magnetic field alignment, thus giving rise to eight possible ESR transitions. The ESR spectrum in Fig. 1C corresponds to a magnetic field almost along one of the four NV axes, thus we observe two transitions well separated from the other six, which lie closer to each other. Our single-crystal diamond has a 〈111〉 face and the magnetic field lies along this direction.

The notable observation in Fig. 1C is that the signal at the highest magnetic field (indicated by a circle in the figure) changes its sign under bright conditions. The ESR signal shows derivative Lorentzian curves due to technical reasons (*35*); therefore, the sign change means whether the Lorentzian starts with an upward or downward direction. The same effect was observed previously in a low magnetic field measurement (at around 1 T) (*29*) which was also reproduced here (data shown in the Supplementary Materials). ESR spectroscopy is based on the phase-coherent detection of microwaves after interaction with the sample (*35*, *36*). In a usual experiment, the microwave radiation is absorbed; therefore, the observed ESR signal phase corresponds to a net microwave photon absorption. The sign reversal in an ESR experiment therefore can only correspond to an extra emission of microwave photons, which is a consequence of population inversion. We therefore unambiguously observe the population inversion at 15 T, corresponding to an emission of 0.42 THz photons. In turn, this observation opens the way to stimulated emission of terahertz radiation.

The data in Fig. 1C allow us to determine the magnitude of the spin polarization (or that of the population inversion) as the ESR transition signal amplitude is directly proportional to the population difference in the initial and the final state. The observed change in the ESR signal in Fig. 1C is a factor of 2 for the lowest transition and a factor of −0.5 for the highest one under the bright conditions. Were the pumping complete, i.e., the population inversion perfect, we would expect a change of a factor of 6 for the lowest and −2.5 for the highest transition as given in the Supplementary Materials. The incomplete saturation of the level populations is the result of a trade-off in our special sample: On one hand, a sample with a high NV concentration is required to be able to perform an ESR experiment at all. On the other hand, it results in a strongly light-absorbing sample, its optical density being 2.7 at 532 nm (see the Supplementary Materials). This means that light-induced pumping is less effective where less optical power is available. This cannot be compensated by increasing the laser power due to sample heating effects.

**Fig. 2.**
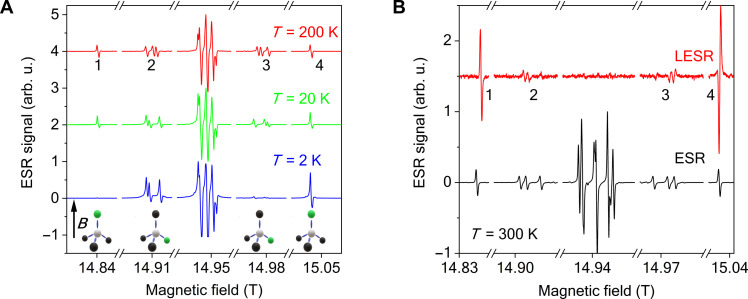
Temperature-dependent electron spin resonance spectra of NV centers and light-induced ESR spectra at room temperature. (**A**) Temperature dependence of the conventional ESR signal at 420 GHz. The intense lines around 14.95 T, i.e., at *g* ≈ 2 correspond to the P1 (charge neutral) centers, while the other eight lines (denoted as 1 to 4) belong to negatively charged NV centers. Note that signals 2 and 4 grow on lowering the temperature, while 1 and 3 vanish. (**B**) The light-induced ESR (LESR) signal at room temperature and 420 GHz together with the conventional ESR data acquired simultaneously shown as reference. Note that the P1 signal is absent in the LESR spectrum and that resonances 1 and 2 have an opposite sign to signals 3 and 4.

To elucidate the nature of the optimal conditions for a population inversion and the resulting terahertz emission, we performed temperature-dependent ESR studies as well as light-induced ESR (LESR) studies (*37*). Figure 2A shows the temperature dependence of the conventional ESR spectra between 200 and 2 K. We introduce a notation for the observed ESR lines: 1 and 4 denote the transitions for the NV center whose axis is parallel to the external magnetic field, whereas 2 and 3 denote the 3-3 transitions for the other three NV centers whose axes have a high angle with the external magnetic field (close to the tetrahedral angle of 109.5°). The exact resonance energies can be obtained by diagonalizing the spin Hamiltonian in a given magnetic field. However, at high magnetic fields (well above the aforementioned *D*/*hg*_e_μ_B_ ≈ 0.1 T), when the DC magnetic field is along one of the NV axes and the other three NV orientations form the tetrahedral angle with it, the resonance frequencies simplify to

f1=1h(B′+D)→B1=B0−D′ (2a)

f2=1h(B′+34D)→B2=B0−34D′ (2b)

f3=1h(B′−34D)→B3=B0+34D′ (2c)

f4=1h(B′−D)→B4=B0+D′ (2d)

where *f*_2_ and *f*_3_ are triply degenerate resonances. We introduced the magnetic field in energy units (*B*′ = *g*_e_μ_B_*B*) and *h* is Planck’s constant. As in continuous-wave ESR, the irradiation frequency (*f*_0_) is constant and the external magnetic field is swept; the resonance frequencies can be rewritten to resonant fields in the high-field indicated by the arrow.

There, we introduced $B_{0}=\frac{h}{g_{e}\mu_{B}}f_{0}$ and the ZFS parameter in magnetic field units *D*′ = *D*/*g*_e_μ_B_.

**Fig. 3.**
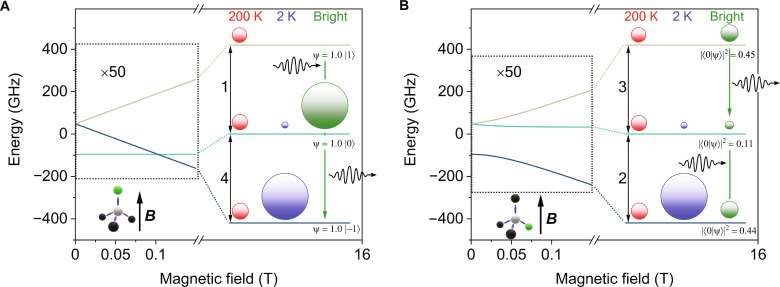
Energy level schemes of the ground state triplet level of NV centers. The magnetic field dependent energy level schemes when the magnetic field is parallel (**A**) or tetrahedral (**B**) to the NV direction. The circle symbol sizes indicate the population of the respective levels for both dark [200 K (red) and 2 K (blue)] (Bright, green) conditions. The incident wavy arrow for the population under illumination indicates the net absorption of microwave photons and the outgoing indicates a net emission of such. The transition labeling is the same as used in Fig. 2. In the parallel case, the ZFS spin states are eigenstates. The full wave function of the tetrahedral case with complex weights is given in equation 2 in the Supplementary Materials. As the population is set by the spin polarization mechanism preferring the |0⟩ state, the square of the weight of |0⟩ is given in (B).

The resonant fields, given on the right side of Eq. 2 (a to d), are numbered in the same order as in Fig. 2A and they also correspond to the notation in Fig. 3. The ESR spectra presented in Fig. 2A change with decreasing temperature: Signals 2 and 4 increase, while signals 1 and 3 vanish. This indicates that signals 2 and 4 correspond to transitions between the lowest and the middle states, whereas signals 1 and 3 correspond to transitions between the middle and highest states.

The LESR data unambiguously identify the light-sensitive transitions. Technical details are given in Materials Methods and we only briefly discuss it here. The LESR method detects variations in the conventional ESR spectra under the action of an amplitude-modulated (or chopped) laser light using a double lock-in technique. The conventional ESR is detected with a magnetic field modulation technique using a lock-in amplifier at around 20 kHz, whereas the laser modulation frequency is three orders of magnitude lower (at around 20 Hz). A second lock-in amplifier detects the variation in the conventional ESR in phase with the optical chopping. The output signal is thus proportional to the difference between the ESR spectra acquired with and without illumination. This is similar to the data shown in Fig 1C, but the lock-in technique allows us to measure much smaller changes in a phase-sensitive manner. While this method shows some resemblance to the more common optically detected magnetic resonance on NV centers (*38*), no such experiments have been performed on this material to our knowledge, the reason probably being the lack of diamond samples with sufficiently large NV concentrations for this study.

Compared with Fig. 1C, where the conventional ESR is shown with and without illumination, Fig. 2B shows the same effect more markedly: (i) the increased intensity of the NV resonance at the lowest field value (signal 1) appears as a (derivative) Lorentzian with a positive phase, (ii) whereas the decreased intensity of the highest NV resonance (signal 4) translates as a line with negative phase in the LESR spectrum. These data reinforce the earlier observation of a light-induced emission for this signal. The P1 resonances of the neutral nitrogen atoms are missing from the LESR spectrum as they do not show optical activity. We performed similar studies in low-field ESR (at ∼9.8 GHz) and the key results are shown in the Supplementary Materials.

However, somewhat unexpectedly, we also observe that signals 1 and 2 as well as 3 and 4 behave similarly in a pairwise manner. We established above that signals 2 and 4 correspond to the lowest state to middle state transition, whereas 1 and 3 correspond to a middle state to the highest state transition. Clearly, the effect of population inversion and thus the emission is also reduced for signals 2 and 3 with respect to signals 1 and 4.

These seemingly contradicting observations can be well explained by considering the actual level mixing for the different geometries as shown in Fig. 3. We show the energy level scheme for the two geometries, i.e., when the magnetic field is parallel to or at the tetrahedral angle to the NV axis. For the earlier, the ZFS spin states remain eigenstates of the Zeeman Hamiltonian but a strong admixture is observed for the latter. The calculation shows that the transitions labeled as 2 and 4 correspond to the lowest to middle state transitions, which explains the temperature-dependent ESR data.

The magnitude of light-induced pumping can be obtained after calculating the eigenvectors in a high magnetic field while using the original ZFS basis. When the magnetic field is parallel to the NV axis, the middle state is populated as it has a purely *S_z_* = 0 character, while the other two levels are the *S_z_* = ±1 states. This explains why transition 4 shows emission, while transition 1 remains of the usual absorptive character. However, the effect is reversed for the other geometry: the lowest and highest states have a stronger *S_z_* = 0 character than that of the middle one as represented by the circle sizes in Fig. 3B. The three states are expressed in the ZFS basis in equation 2 in the Supplementary Materials, and Fig. 3B shows the absolute square of the probability amplitude of the corresponding states being in the ∣0〉 state. The intensity of an ESR transition is proportional to the population difference between the final and initial states and the population is driven by the spin polarization preferring the ∣0〉 state. This means that upon light pumping, transition 3 has an emission character although weaker than that of transition 4.

A detailed calculation of the population of states at different temperatures and also under illumination is given in the Supplementary Materials. The ESR intensity is proportional to the population difference of the corresponding states, whereas the LESR signal is proportional to the change between the bright and dark population differences. The intensity ratios of LESR lines given by our model in 15 T external magnetic field read

$$I_{1}:I_{2}:I_{3}:I_{4}=0.94:0.27:-0.37:-1.00$$ (3)

where *I*_2_ and *I*_3_ refer to the intensity of one of the three possible tetrahedrally lying NV centers, i.e., in a perfect alignment the measured intensity should be three times this value due to the threefold degeneracy. However, in our case, there is a slight misalignment lifting the degeneracy, so the intensity values represented here refer to one ESR line (here, the average of the three), not the sum of three. The phase of the observed LESR signal matches the predictions of the simplified model but the intensity ratio obtained from Fig. 2B is

$$I_{1}:I_{2}:I_{3}:I_{4}=0.58:0.09:-0.13:-1.00$$ (4)

It differs from the calculated values in Eq. 3, namely, the inner lines are less intense than predicted by the wave functions.

This is a manifestation of the polarization dependence of the electric dipole transition. The optical excitation of NV centers depends on the relative angle between the laser polarization and the NV center axis (*39*, *40*). In our experiment, the sample is placed with its 〈111〉-axis parallel to the direction of propagation of the linearly polarized laser beam, leaving the other three NV directions at a tetrahedral angle with respect to it. As shown in (*40*) in a similar geometry, the pumping rate (*P*) of the NV centers lying at a tetrahedral angle strongly depends on the angle (φ) between the laser polarization and the projection of the NV center on the surface. The effective pumping rate is

$$P=P_{0}\left({\text{sin}}^{2}\varphi +\frac{1}{9}{\text{cos}}^{2}\varphi \right)$$ (5)

where *P*_0_ is the pumping rate of the NV centers in the 〈111〉 orientation. Depending on the rotation angle around the 〈111〉-axis, this factor varies between *1* and 1/9 continuously. However, for the three possible orientations denoted by angles φ, φ − 120°, and φ + 120°, the sum (*P*_φ_ + *P*_φ−120°_ + *P*_φ+120°_) is always $\frac{5}{3}P_{0}$ . With this correction, the theoretical values of *I*_2_ and *I*_3_ are 0.15 and −0.2, respectively. To explain the remaining difference and the experimentally lower value of *I*_1_, the insufficient optical power (due to the sample optical density mentioned earlier) should be included in a more sophisticated model.

Aside from the better sensitivity to detect the optically induced changes in ESR, the LESR technique also enables studying spin dynamics. Knowledge of the relevant ESR relaxation times (*T*_1_ and *T*_2_) in the NV centers plays a key role in applications, e.g., quantum technology, metrology, sensing, and the maser applications (*21*, *41*, *42*). These relaxation times are usually measured using pulsed ESR techniques. However, such instruments are not yet available at high frequencies. It would also be possible to measure the relatively long spin-lattice relaxation time, *T*_1_, in NV centers using saturation experiments (*33*, *43*) but the near-THz ESR instruments lack the required absolute power accuracy.

In the LESR technique, *T*_1_ is measurable by staying on a given resonance line (we studied signals 1 and 4, i.e., when the magnetic field is parallel to the NV axis) and varying the frequency of the optical chopping in the 1 to 400 Hz range. During irradiation, the level populations start deviating from their equilibrium values; when the light is switched off, the system recovers to equilibrium with a time constant of *T*_1_.

**Fig. 4.**
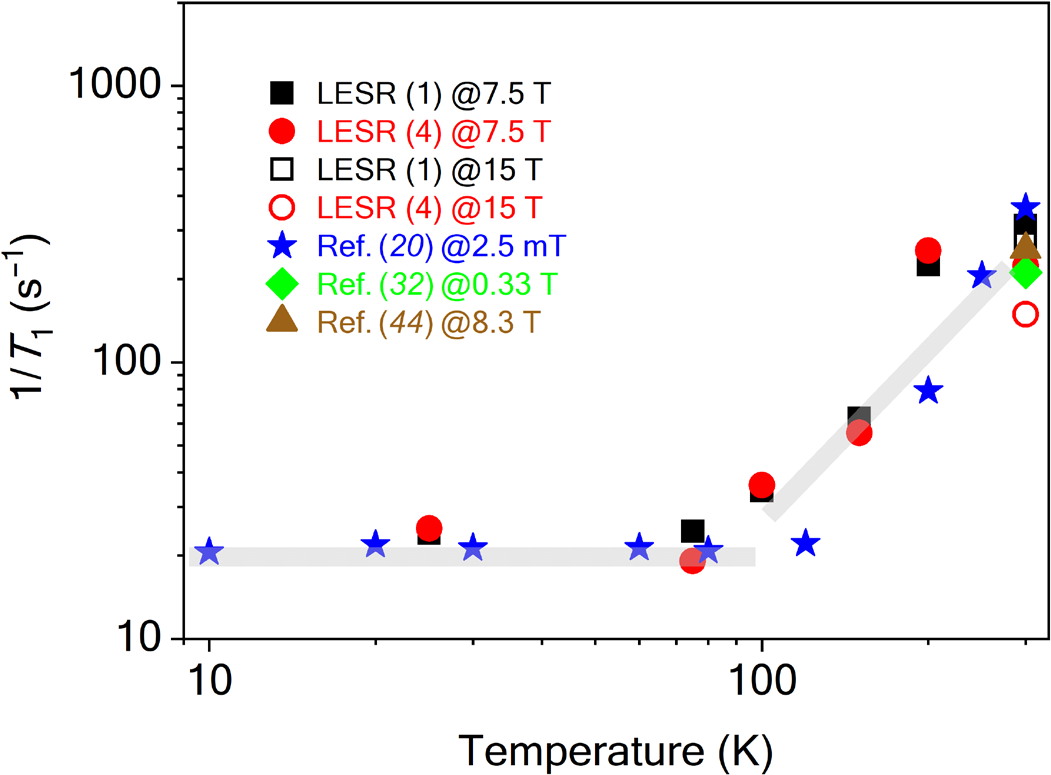
Temperature dependence of spin-lattice relaxation time of NV centers measured by LESR technique. 1/*T*_1_ as a function of temperature measured on the lowermost [LESR (1)] and uppermost lying [LESR (4)] NV center LESR lines at 7.5 T (filled symbols) and also at room temperature for 15 T (open symbols). LESRs (1) and (4) correspond to signals 1 and 4 of Fig. 2, respectively. For comparison, we show previously reported data on diamond samples with similar NV center density: a full temperature dependence at 2.5 mT (*20*) and two room temperature results at 0.33 T (*32*) and 8.3 T (*44*). Gray lines are guide to the eye indicating a residual relaxation and a phonon-dominated region.

In Fig. 4. 1/*T*_1_ values obtained with the double modulation technique are presented and further technical details are given in the Supplementary Materials. Temperature-dependent data are given at 7.5 T and data at room temperature is shown at 15 T (0.42 THz). The lower overall sensitivity of the high-frequency ESR (HFESR) spectrometer at 0.42 THz [it is optimized for 0.21 THz (*30*, *31*)] combined with the extensive measurement time and magnetic field instability limited these measurements further. We include in the figure previously reported experimental data measured on samples that are similar to ours, i.e., HPHT grown, exposed to high-dose electron beam irradiation, resulting in a concentration above 100 ppm of NV centers after high-temperature annealing (*20*).

## DISCUSSION

Our temperature-dependent *T*_1_ results agree well with the results in (*20*), although the latter experiment was performed at *B* = 2.5 mT, i.e., in a 6000 times smaller magnetic field. Also, pulsed X-band (at 0.33 T) ESR measurements (*32*) show a *T*_1_ relaxation time of about 4.73 ms at room temperature, while high-field optically detected magnetic resonance measurements yield 3.9 ms (*44*). These results are in good agreement with our high-field LESR results at 7.5 T and at 15 T. This indicates that the spin-lattice relaxation is unaffected by the high magnetic fields, which is important for the future use of the NV center in the aforementioned applications.

Our measurement does not directly determine the *T*_2_ (spin decoherence time) and $T_{2}^{*}$ (spin dephasing time) albeit both are relevant for spintronics applications, especially to those where the interplay between coupled spin-polarized carriers and photons is exploited (*45*, *46*). We nevertheless do not observe a substantial line broadening although the applied magnetic field is 50 times larger than the customary 0.3 T in X-band. This is expected for *T*_2_ as it is caused by spin-spin interactions (both like and unlike spin-spin interactions), described by the van Vleck formula (*36*). The $T_{2}^{*}$ could, in principle, be caused by magnetic field inhomogeneity, but for solid-state systems, the dephasing time is dominated by impurities or by unresolved hyperfine interactions (*47*), both of which are independent of the magnetic field. We, therefore, expect that both *T*_2_ and $T_{2}^{*}$ can be well estimated by pulsed ESR experiments in low fields, where such measurements are possible (*32*). This is very promising for the expected TASER applications as the cooperativity factor would be limited by a short $T_{2}^{*}$.

A conveniently long *T*_1_ is required to preserve a high level of population inversion as, otherwise, this process would deplete the inverted population. This potentially makes the diamond NV center in a high magnetic field a possible candidate for a coherent terahertz source (a TASER) or terahertz amplifier with ample technical possibility for magnetic field tuning and modulation, which may be eventually advantageous for conventional and quantum communication and various condensed matter applications. We outline and critically assess the possible construction of a diamond NV–based TASER in the Supplementary Materials. However, exploiting the true capabilities of this system requires substantial additional work to properly describe the TASER-relevant cooperativity factor (*21*) in a high magnetic field.

In summary, we showed that the diamond NV centers can operate as sources of coherent terahertz emission when subjected to a high magnetic field. This effect is achieved by pumping the sample with visible light and achieving a population inversion. The dependence of the pumping efficiency on the respective orientation of the NV axis and the magnetic field was observed using a sensitive method, light-induced ESR. The observation was explained by considering the spin Hamiltonian of the NV center and also by the variation of the light polarization with respect to the NV axis. The LESR technique was also used to determine the spin-lattice relaxation time, *T*_1_, at 7.5 T and at 15 T, and it showed that the dominant relaxation mechanisms are unaffected by the magnetic field.

## MATERIALS AND METHODS

**Fig. 5.**
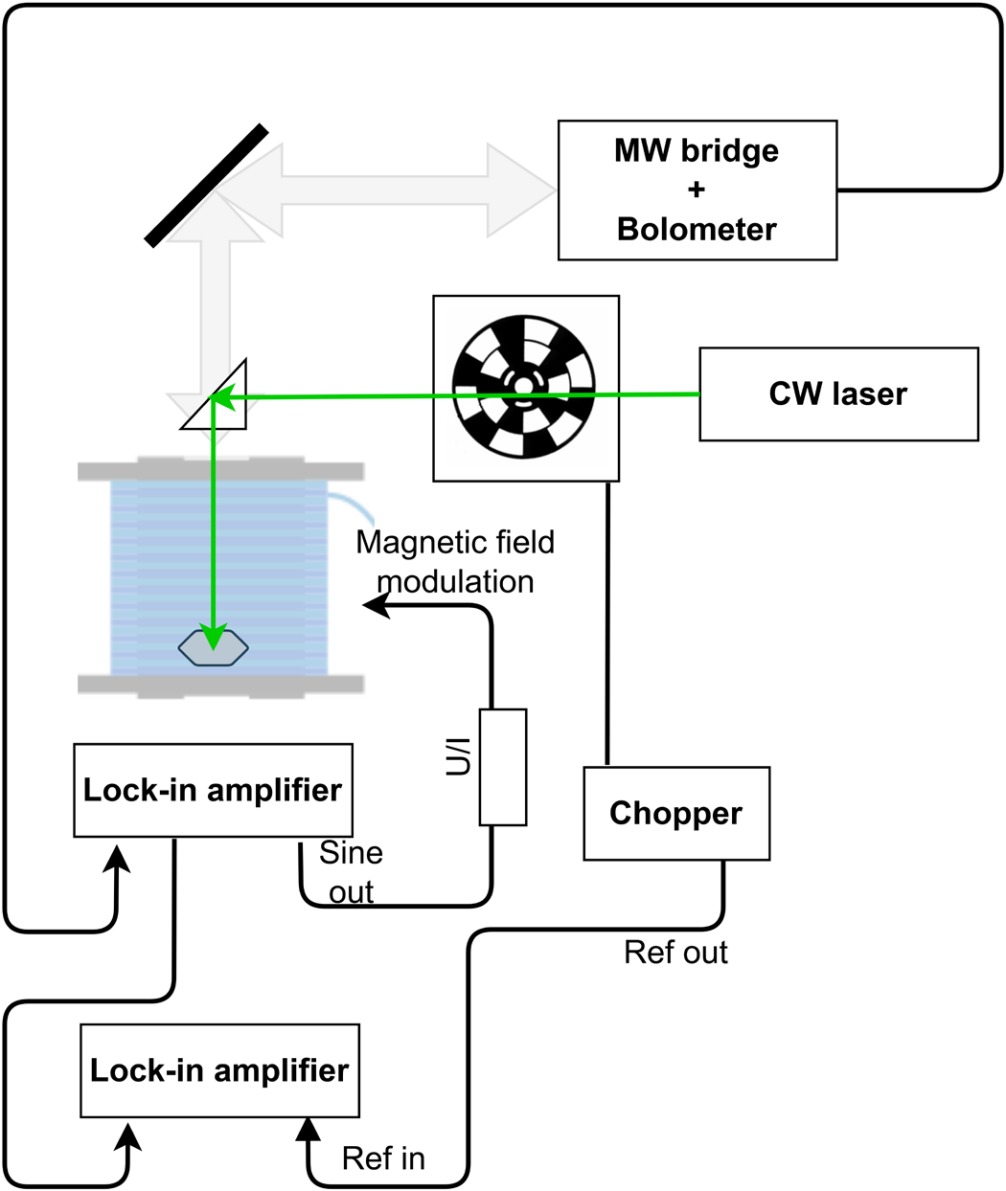
High-field/high-frequency ESR setup with optical excitation. Microwaves are directed through a corrugated waveguide and the reflected waves are detected using a bolometer. The signal from the bolometer is fed into a lock-in amplifier which modulates the external magnetic field. Its down-mixed output is fed into a second lock-in amplifier, controlling the visible laser chopping, eventually producing the light-induced ESR signal.

All experiments presented here were carried out on a single crystal diamond plate (type 1b produced by high-pressure/high-temperature method, supplied by Element Six Ltd.) of a hexagonally cut shape (diameter about 5 mm) with NV center concentration of about 12 ppm which was produced by electron beam irradiation and subsequent thermal annealing. More details on sample preparation and quantitative analysis are given in (*32*) and in the Supplementary Materials. The block diagram of the high-frequency light-induced ESR spectrometer is shown in Fig. 5. It is based on an HFESR spectrometer operating in the 0.052 to 0.420 THz frequency range described in (*30*). This instrument works with phase-locked loop stabilized, frequency-multiplied microwave (of near-terahertz) sources at a number of discrete frequencies. Detection is based on a liquid helium-cooled InSb hot-electron bolometer used in a homodyne single-ended mixer configuration. The detector is part of a millimeter-wave, quasi-optical bridge, which allows for the phase-sensitive detection of reflected radiation from the sample.

The sample is placed on a piezo-controlled single-axis goniometer (attocube systems AG) and is inside a liquid helium variable temperature insert (VTI) inside a superconducting solenoid with fields up to 16 T (Oxford Instruments). The sample chamber is isolated from the VTI and is embedded in helium exchange gas. Microwaves propagate back and forth to the sample from the top in a corrugated waveguide which is covered by a 1-inch (2.54-cm) clear diameter polyethylene (PE) window. Optical access to the sample was also obtained from the top. The original PE window was first replaced by a 5-mm-thick BK7 glass but it caused more than 50% decrease in microwave power reaching the sample. We found that a thin (≈300 μm) sheet of Mylar (also known as BoPET i.e., biaxially oriented polyethylene terephthalate) is a good compromise, excellent for vacuum isolation and it causes less than 10% microwave loss for our frequency range, while it is transparent in the visible-light range resulting in only 5 to 10% of optical power loss.

The sample is at the bottom of a 2-m-long corrugated waveguide that efficiently directs the millimeter waves toward the sample. The waveguide ends in a hollow brass tube with an approximate diameter of 5 mm. The system is equipped with a frequency-doubled Nd:YAG laser working at λ = 532 nm with a maximum output power of 150 mW. Guiding the light was achieved by constructing a precise optics breadboard with four mirrors and pinholes, which were fixed to the probe head. The light passes through a Mylar window that introduces a 5 to 10% loss in the visible spectrum. The main loss in optical power reaching the sample is caused by the beam divergence and the resulting reflections from the walls of the waveguide. After careful alignment of the mirrors, the maximum power passing through the brass tube was 20 mW. This incident power equals an intensity of 1 W/cm^2^.

A magnetic field, parallel to the DC magnetic field, is modulated with an amplitude of 0.05 mT at 20 kHz, which is customary in ESR spectroscopy. The bolometer output signal is detected phase-sensitively with a lock-in amplifier (Stanford Research Systems, SR830). This output provides the conventional HFESR signal. To obtain the light-induced ESR signal, the laser light is modulated with an optical chopper (Stanford Research Systems, SR540). Then, the demodulated output signal from the first lock-in amplifier is fed into a second lock-in amplifier (SR830) which controls the chopper. The second demodulated output produces the light-induced ESR signal as demonstrated in (*37*, *48*).

## Supplementary Materials

### This PDF file includes:

Supplementary Materials and Methods Supplementary Text Figs. S1 to S9 Tables S1 to S4 References
