# Supplementary material for: Terahertz emission from diamond nitrogen-vacancy centers
Source: Sci Adv. 2024 May 29;10(22):eadn0616. doi: 10.1126/sciadv.adn0616 (PMC11135399; doi:10.1126/sciadv.adn0616)
Supplement: Supplementary file 1 — Supplementary Materials and Methods Supplementary Text Figs. S1 to S9 Tables S1 to S4 References [file sciadv.adn0616_sm.pdf]

Science Advances
AAAS

Supplementary Materials for

Terahertz emission from diamond nitrogen-vacancy centers

Sándor Kollarics *et al.*

Corresponding author: Ferenc Simon, simon.ferenc@ttk.bme.hu

Sci. Adv. **10**, eadn0616 (2024)
DOI: 10.1126/sciadv.adn0616

This PDF file includes:

Supplementary Materials and Methods
Supplementary Text
Figs. S1 to S9
Tables S1 to S4
References

1 Sample preparation

Type 1b single crystal diamond produced by the high pressure high temperature method was purchased from Element Six Ltd (Didcot, UK). The crystal was exposed to electron irradiation in a radio frequency linear accelerator with variable energy between 1 MeV and 4 MeV built by RadiaBeam Technologies LLC (Santa Monica, CA, USA). The sample received a total electron

fluence of $2.18 \cdot 10^{18}$ $1/\mathrm{cm}^2$ determined from the resulting current of the accelerator. Subsequently, the sample was annealed at 800 °C for two hours and then at 1000 °C for two hours again. NV center concentration was determined first relative to nitrogen concentration and then calibrated against copper(II) sulfate pentahydrate, $\mathrm{CuSO_4 \cdot 5H_2O}$ standard in a conventional X-band continuous wave electron spin resonance (CW ESR) spectrometer (Bruker Elexsys E500). The ratio of NV centers compared to neutral substitutional nitrogen atoms in the diamond lattice was found to be 13.2% yielding 12 ppm NV concentration considering 90 ppm nitrogen concentration based on the CW ESR spectra.

2 Population of states in high and low magnetic field

In this section, we show the calculation behind the statements in the main text related to the population of the eigenstates in a finite magnetic field. We diagonalize the spin Hamiltonian in two cases: (i) when the external magnetic field $\boldsymbol{B}$ is parallel with the NV-axis and (ii) when $\boldsymbol{B}$ forms a tetrahedral angle with the NV-axis, i.e., the orientation of the other three NV-s in case (i). The resulting energies are expressed in temperature (K) for convenience. Without illumination, the population is described by the Boltzmann distribution, where the probability of a particle occupying state *i* is:

$$p_i = \frac{N_i}{N} = \frac{\exp\left(-\frac{\varepsilon_i}{k_\mathrm{B}T}\right)}{\sum_i \exp\left(-\frac{\varepsilon_i}{k_\mathrm{B}T}\right)}. \tag{1}$$

The LESR experiments presented in Fig. 2 in the main text were carried out at 200 K and in 15 T so we use these values in our present calculation. During illumination, the spin polarization effect competes with the Boltzmann distribution. The spin polarization is described with a set of differential equations (rate equations) introducing several transition probabilities (*49*). In high magnetic field and at a finite temperature, the exact populations could only be given with a system of differential equations using additional input parameters such as absorbed optical

power and spin-lattice relaxation time aside from the already complicated spin polarization dynamics. Our aim is to understand the light-induced changes to the ESR signal in high magnetic fields and to qualitatively explain the LESR signal presented in Fig. 2 in the main text. For that, we investigate the high optical intensity regime where the effect of finite temperature can be neglected. So in our simplified model, the population of a given state is proportional to the $S_z = 0$ coefficient. In the parallel case, the finite magnetic field eigenstates are pure S_z states therefore when illuminated the middle state is fully populated whereas in a tetrahedral case, the population depends on the strength of the magnetic field. Whereas in the tetrahedral case, the eigenstates are linear combinations of the pure ZFS states such as shown here in Eq. (2) in an external magnetic field of 15 T:

$$\begin{aligned}\Psi_{\mathrm{U}} &= (-0.5761 - 0.3326\,\mathrm{i})\,|{-1}\rangle + (0.5786 + 0.3340\,\mathrm{i}))\,|0\rangle \\ &\quad +(0.2887 + 0.1667\,\mathrm{i})\,|1\rangle\,, \\ \Psi_{\mathrm{M}} &= (0.1729 - 0.6453\,\mathrm{i})\,|{-1}\rangle + (0.0863 - 0.3220\,\mathrm{i})\,|0\rangle \\ &\quad +(0.1722 - 0.6426\,\mathrm{i})\,|1\rangle\,, \\ \Psi_{\mathrm{L}} &= 0.3333\,|{-1}\rangle + 0.6652\,|0\rangle - 0.6681\,|1\rangle\,.\end{aligned} \tag{2}$$

Diagonalizing the spin Hamiltonian in 15 T with ZFS of 0.1 T we acquire eigenvalues of the lowest, middle and upper states -20.14 K, -0.09 K and 20.23 K in the parallel and -20.20 K, 0.03 K and 20.17 K. in the tetrahedral orientation. Substituting this into equation (1), we can calculate the population of the given states.

The ESR intensity is proportional to the population difference. Therefore the CW ESR intensity ratios of NV centers in 15 T in the strong illumination limit would yield:

$$I_1 : I_2 : I_3 : I_4 = 1 : 0.32 : -0.35 : -1 \tag{3}$$

The LESR measures the change induced by the light, therefore the intensity is proportional

$B = 15$ T parallel case	Temperature			
State	2 K	20 K	200 K	Bright
U	0	0.08876	0.30024	0
M	0.00004	0.24299	0.33033	1
L	0.99996	0.66825	0.36740	0

$B = 15$ T tetrahedral case	Temperature			
State	2 K	20 K	200 K	Bright
U	0	0.08876	0.30033	0.46
M	0.00004	0.24299	0.33215	0.11
L	0.99996	0.66825	0.36751	0.43

Table S1: **Population of states at given temperatures and under illumination in 15 T.**

$B = 15$ T parallel case	Temperature				
State	2 K	20 K	200 K	Bright	Bright-Dark
M-U	0	0.1561	0.0321	1	0.9679
L-M	0.9999	0.4221	0.0350	-1	-1.035

$B = 15$ T tetrahedral case	Temperature				
State	2 K	20 K	200 K	Bright	Bright-Dark
M-U	0	0.1542	0.0318	-0.35	-0.3818
L-M	0.9999	0.4253	0.0354	0.32	0.2846

Table S2: **Population difference between states at given temperatures and under illumination in 15 T.**

to the difference in the population difference in the bright and dark measurements:

$$I_1 : I_2 : I_3 : I_4 = 0.9679 : 0.2846 : -0.3818 : -1.035 \tag{4}$$

Diagonalizing the spin Hamiltonian in 0.35 T with ZFS of 0.1 T we acquire eigenvalues of the lowest, middle and upper states -0.48 K, 0.001 K and 0.47 K in the parallel and -0.49 K, 0.03 K and 0.46 K in the tetrahedral orientation. Substituting this into equation (1), we can calculate the population of the given states.

The CW ESR intensity ratios of NV centers in 0.355 T in the strong illumination limit:

$$I_1 : I_2 : I_3 : I_4 = 1 : 0.25 : -0.42 : -1 \tag{5}$$

The LESR intensity ratios in 0.355 T in the strong illumination limit:

$$I_1 : I_2 : I_3 : I_4 = 0.9995 : 0.2494 : -0.4205 : -1.0006 \tag{6}$$

3 Dark and bright room temperature CW ESR spectra

In Fig. S1 room temperature X-band ($B_0 \approx 0.35$ T) ESR spectra are shown measured with and without illuminating the diamond sample. In the dark spectrum, the part with resonant lines of NV centers is scaled up with a factor of 200, whereas the bright spectrum is the raw data. The data presented in Fig. S1 is in qualitative accordance with the intensity ratios calculated in Eq. (5), namely the sign of the intensities resembles the sign of the resonances. The model predicts less intense inner lines but the measurement shows even smaller results.

4 Determining T_1 from the double-modulated LESR signal

Without illuminating the sample, the spin population of the system is given by the Boltzmann equilibrium, while illumination leads to the spin-polarized steady-state. Switching the illumination on and off, the system starts to relax to its proper steady-state enabled by the spin-lattice

$B = 0.35$ T parallel case	Temperature 300 K	
State	Dark	Bright
U	0.3328	0
M	0.3333	1
L	0.3339	0

$B = 0.35$ T tetrahedral case	Temperature 300 K	
State	Dark	Bright
U	0.3328	0.5314
M	0.3333	0.1084
L	0.3339	0.3602

Table S3: **Population of states at room temperature in 0.35 T.**

relaxation. Therefore, the input voltage of the second lock-in amplifier follows a single exponential decay with the spin-lattice relaxation time as time constant. The phase-sensitive detection of the second lock-in can be mathematically described as multiplying the input voltage with the reference signal (with local oscillator frequency of $f = 2\pi\omega$) and integrating it over a full

$B = 0.35$ T parallel case	Temperature 300 K		
State	Dark	Bright	Bright-Dark
M-U	0.0005	1	0.9995
L-M	0.0006	-1	-1.0006

$B = 0.35$ T tetrahedral case	Temperature 300 K		
State	Dark	Bright	Bright-Dark
M-U	0.0005	-0.4235	-0.4235
L-M	0.0006	0.2513	0.2507

Table S4: **Population difference at room temperature in 0.35 T.**

period:

$$X = \int_0^{\frac{2\pi}{\omega}} \cos(\omega t) \mathrm{e}^{-t/T_1} \, \mathrm{d}t = T_1 \frac{1}{1 + \omega^2 T_1^2} \left(1 - \mathrm{e}^{\frac{2\pi}{\omega T_1}}\right) \tag{7}$$

$$Y = \int_0^{\frac{2\pi}{\omega}} \sin(\omega t) \mathrm{e}^{-t/T_1} \, \mathrm{d}t = T_1 \frac{\omega T_1}{1 + \omega^2 T_1^2} \left(1 - \mathrm{e}^{\frac{2\pi}{\omega T_1}}\right). \tag{8}$$

Consequently, the magnitude of the LESR signal:

$$\begin{aligned} R &= \sqrt{X^2 + Y^2} \\ &= T_1 \left(1 - \mathrm{e}^{\frac{2\pi}{\omega T_1}}\right) \sqrt{\left(\frac{1}{1 + \omega^2 T_1^2}\right)^2 + \left(\frac{\omega T_1}{1 + \omega^2 T_1^2}\right)^2} \\ &= T_1 \left(1 - \mathrm{e}^{\frac{2\pi}{\omega T_1}}\right) \frac{1}{\sqrt{1 + \omega^2 T_1^2}}. \end{aligned} \tag{9}$$

Therefore, by sweeping the modulation frequency and fitting the acquired LESR magnitude with

$$S(f) = \alpha \left(1 - \mathrm{e}^{\frac{1}{f\beta}}\right) \frac{1}{\sqrt{1 + (2\pi f)^2 \beta^2}} \tag{10}$$

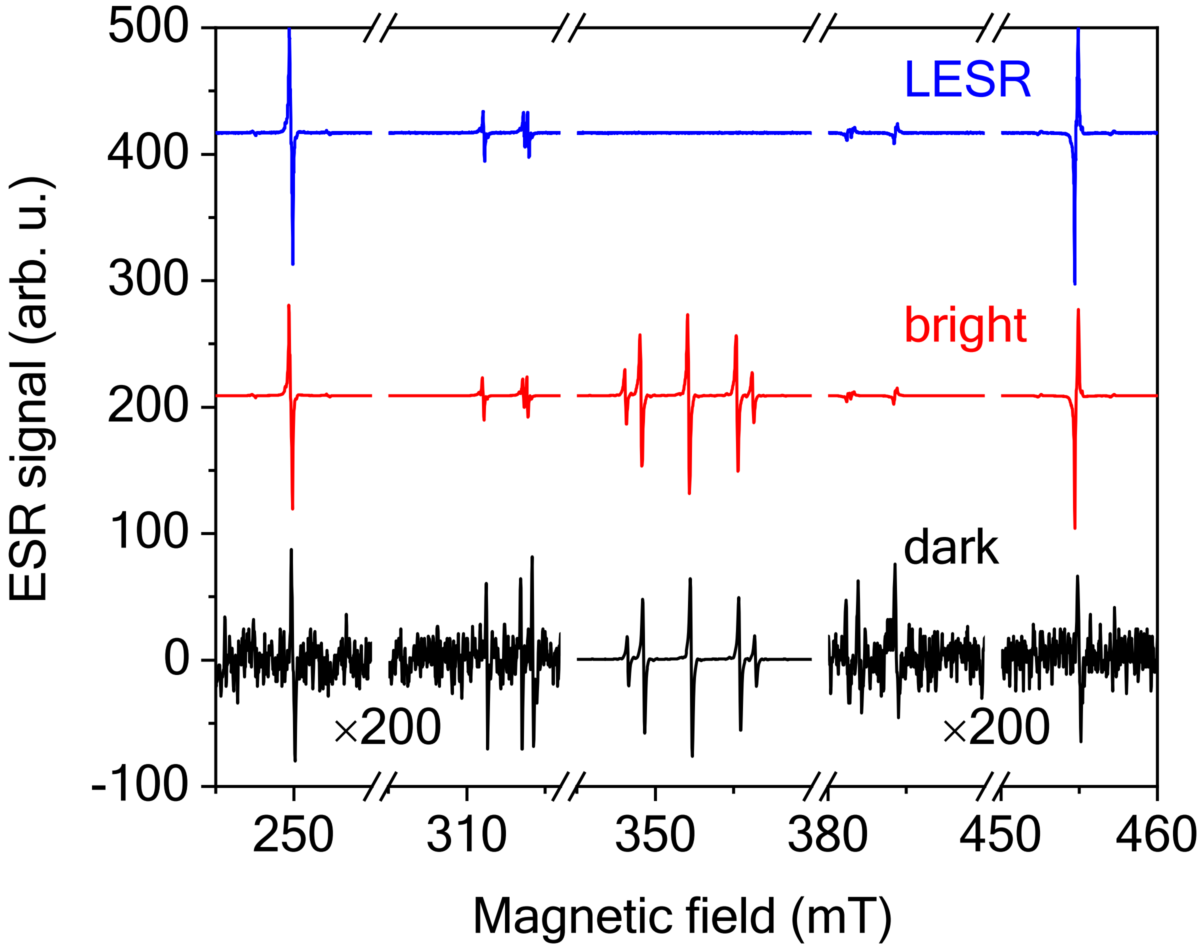

Figure S1: **X-band ($f \approx 9.8$ GHz) light-induced ESR**. Resonances of NV centers are scaled up with a factor of 200 for clarity in the spectrum acquired without illumination (black line). Under continuous illumination, the lower four resonances grow in intensity and the upper four resonances reverse sign as a clear indication of population inversion.

one yields the spin-lattice relaxation time as β as shown in Fig. S2. The parameter α is proportional to T_1 but also to the steady-state magnetization. Note that in the above derivation, $\alpha = T_1$ holds only because the amplitude of the input signal in Eq. (7) and in Eq. (8) is set to one.

5 Additional details of the LESR technique

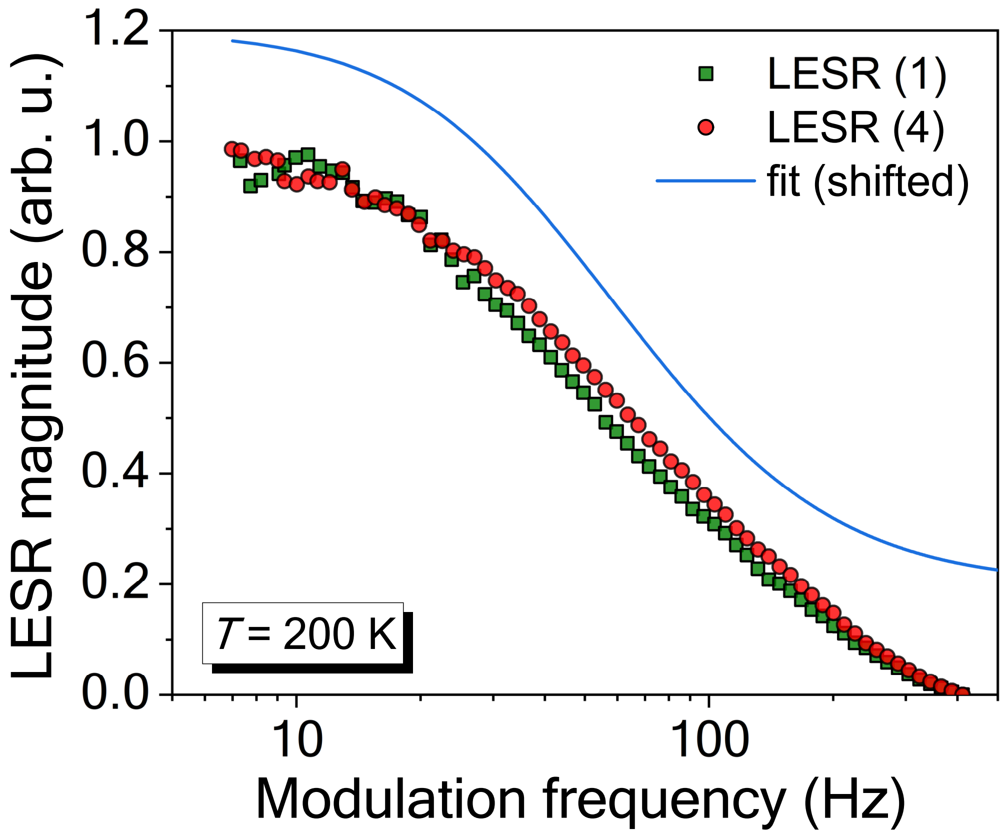

Figure S2: **LESR magnitude of Signal 1 (green squares) and 4 (red circles) as a function of the chopping modulation frequency that was swept in the $1-400$ Hz range at 200 K**. The fitted low-pass characteristic, described by Eq. (10), is shown with a straight blue line, shifted for better visibility.

Figure S3 shows LESR spectra acquired with the same magnetic field sweep velocity but with two different optical modulation frequencies. In both cases, the optical modulation is far from the 20 kHz magnetic field modulation as the principles of double modulation technique require it (*50*), but the second modulation frequency (the optical chopping) interferes with the sweeping resulting in spectral leakage.

Spectral leakage occurs when f_{chop} and f_{sweep} are not separated sufficiently. The latter, f_{sweep} is obtained from the linewidth, ΔB (in units of Tesla), and the field sweep speed, sw (in units

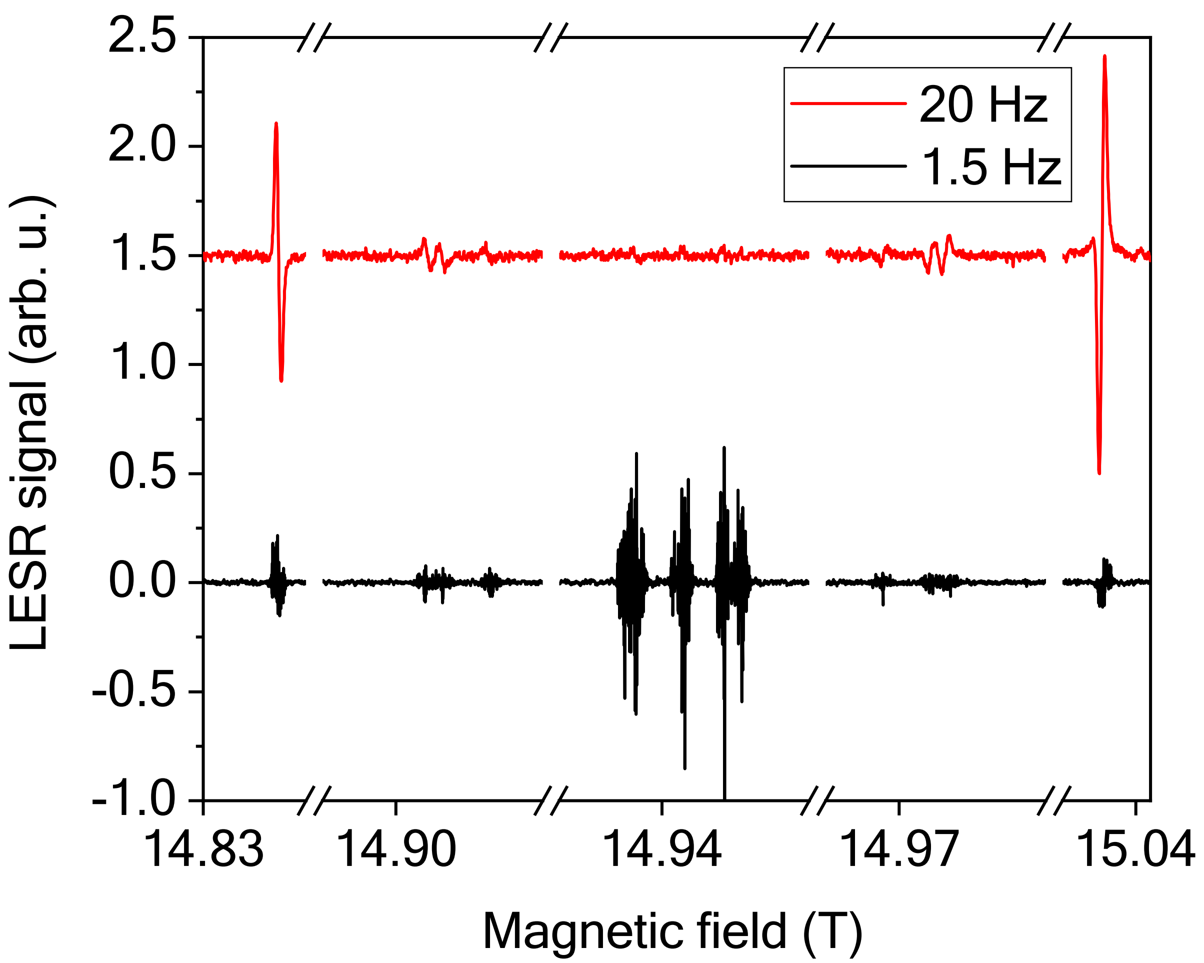

Figure S3: **Light-induced ESR signal of NV centers**. Both spectra were acquired with the same magnetic field sweep velocity of $0.004\,\mathrm{T/min}$ but with different optical modulation frequencies.

of Tesla/sec) as $f_{\text{sweep}} = sw/\Delta B$. The typical linewidths of the observed ESR lines are about 0.1 mT and we used field sweeps between $0.07 - 0.7$ mT/sec, we obtain $f_{\text{sweep}} = 0.7\ldots7$ Hz. This explains why we observe a residual LESR signal for the P1 line when either f_{chop} is varied and when it is too small compared to f_{sweep} (as shown in Fig. S3), or when f_{sweep} is varied and it is too large compared to f_{chop} (as seen in Fig. S4).

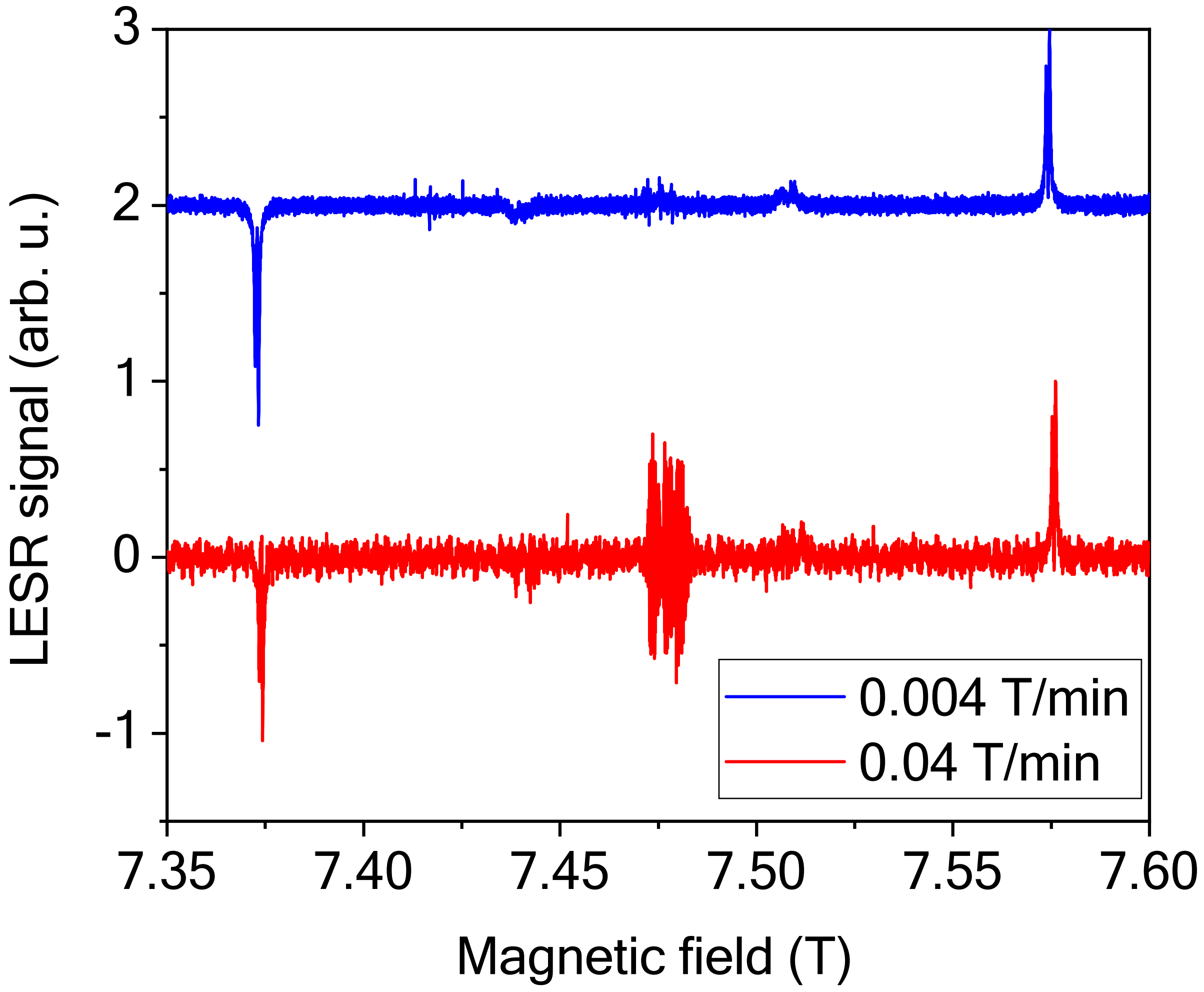

Figure S4: **Light-induced ESR signal of NV centers**. Both spectra were acquired with 40 Hz optical modulation but with different magnetic field sweep velocities.

6 Angular dependence of the high field ESR of NV centers

The resonance NV spectra strongly depend on the direction of the magnetic field with respect to the four possible NV-axes. Figure S6 shows the angular dependence for a sample rotated along the $\langle 11\overline{2} \rangle$ axis, compiled from 89 individual ESR spectra.

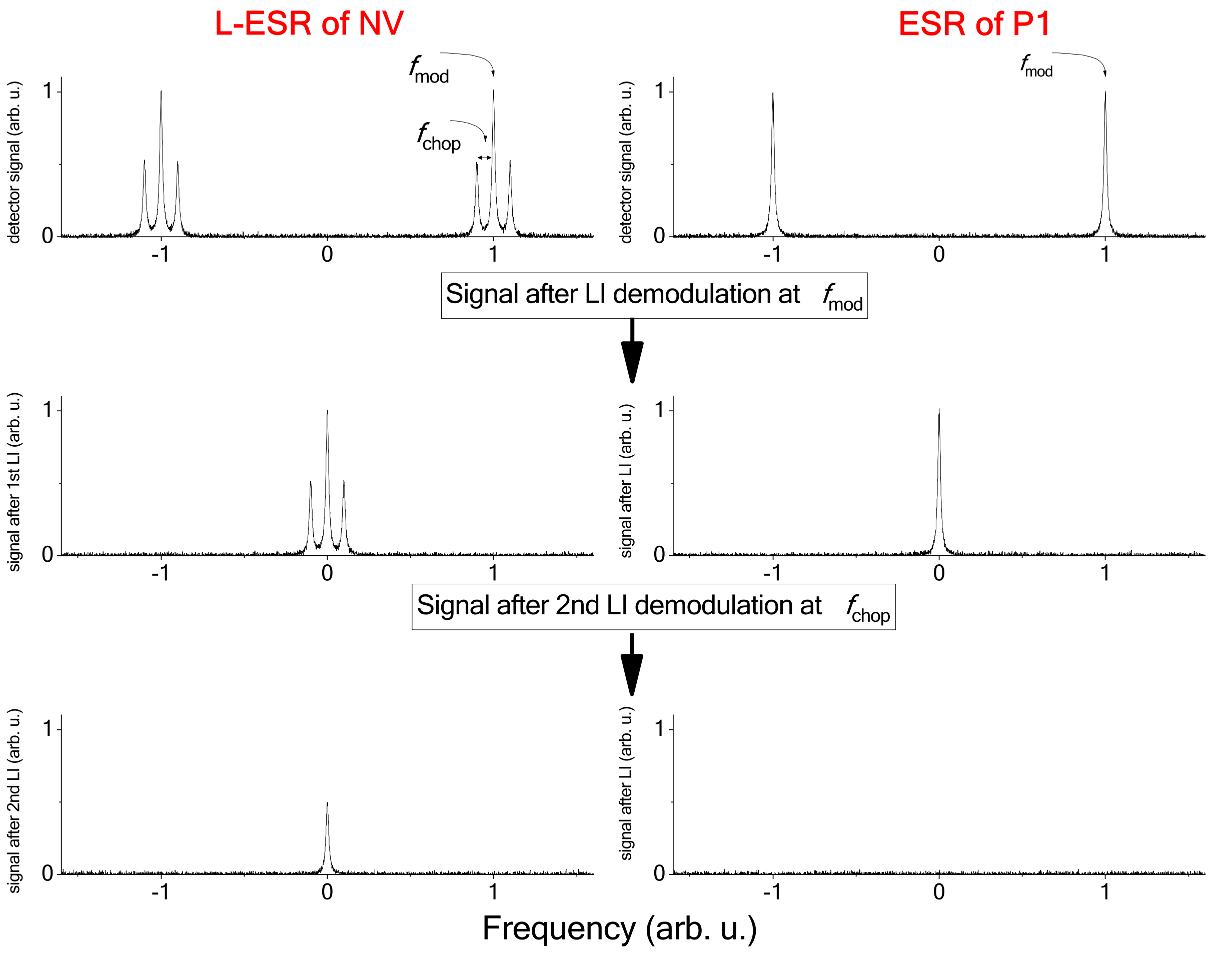

Figure S5: **Schematical explanation of the observed double lock-in detected signals with varying parameters.**

7 Visible absorption of the diamond sample used in our studies

Absorption in the visible range was measured to address the illumination and spin polarization difficulties of NV centers in the HFESR measurements. Figure S7 shows the absorbance with the characteristic feature of the zero-phonon line of NV centers.

The absorption is given as the negative logarithm of the transmission, i.e., the ratio of the

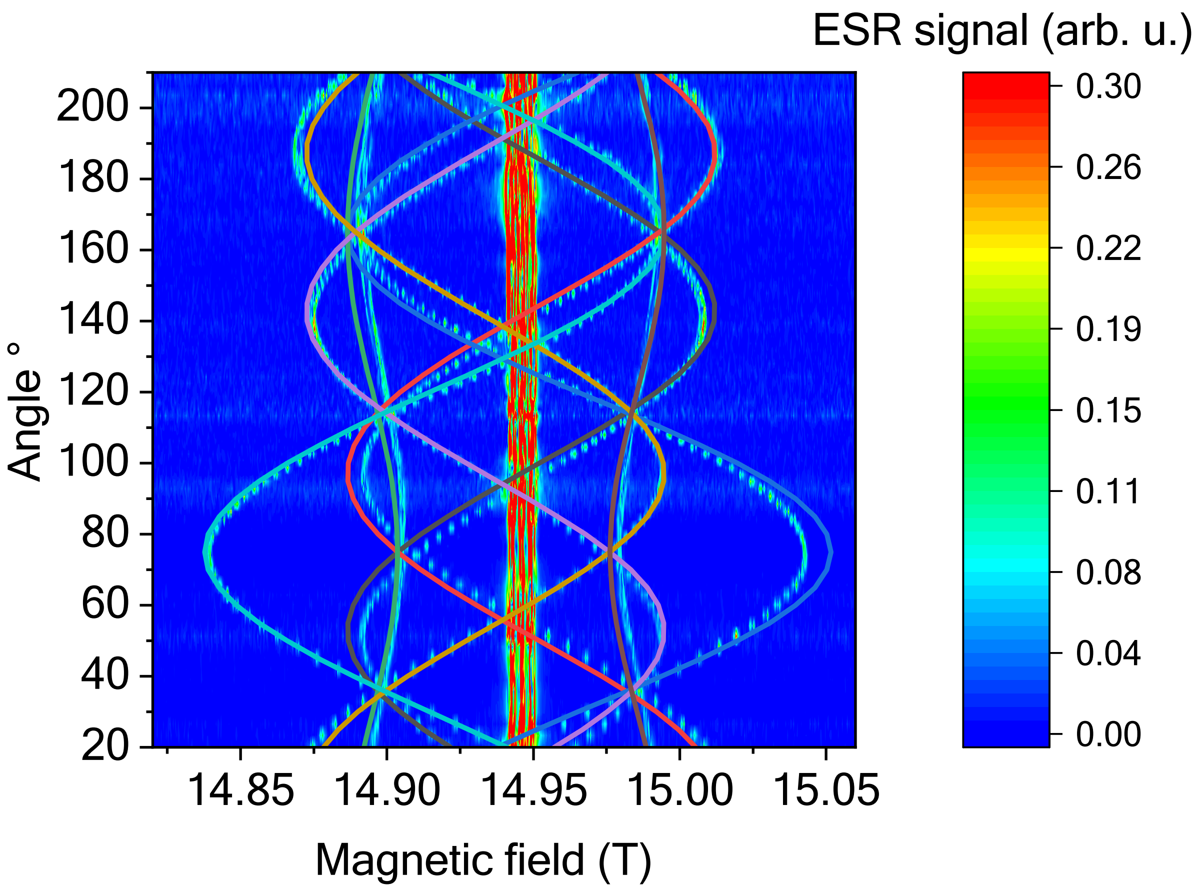

Figure S6: **Angular dependence of the high field ESR of NV centers.** When rotated along the $\langle 11\overline{2} \rangle$ crystalline axis the ESR spectrum shows a significant angular dependence. The strong lines around 14.95 T (the $g = 2$ resonance position) correspond to the P1 ESR signal. Solid lines are simulated resonance positions.

intensity with (I) and without (I_0) the sample being in the beam:

$$A = -\log\left(\frac{I}{I_0}\right). \tag{11}$$

The intensity of the light after passing through the sample is:

$$I = I_0 10^{-A} = I_0 10^{-\alpha d} = I_0 \exp(-\sigma n d), \tag{12}$$

where d is the thickness of the sample, σ is the absorption cross-section and n is the number of NV centers per unit volume. From Eq. (12) a connection between NV center concentration and

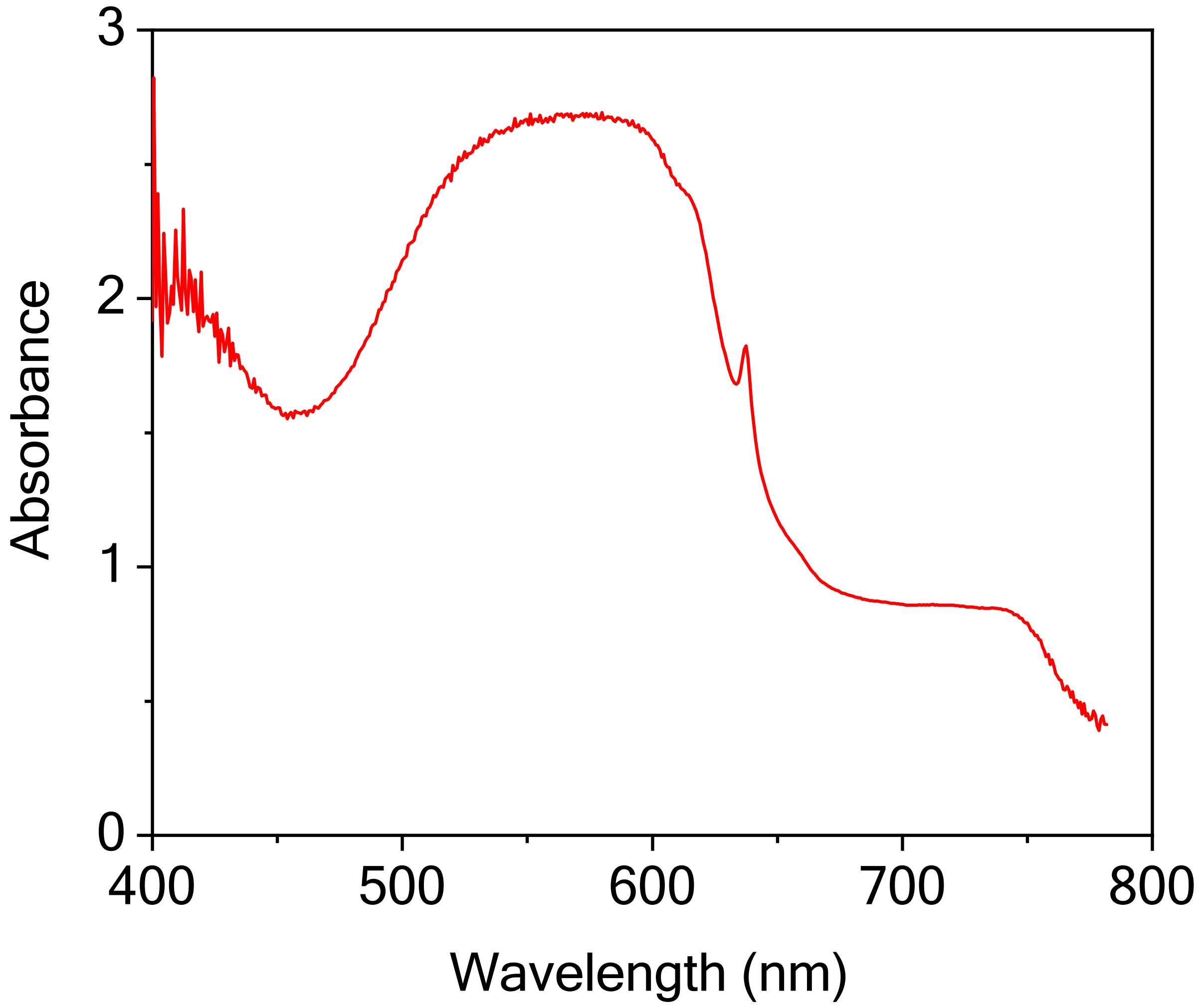

Figure S7: **Visible absorption spectrum of the diamond sample**. Absorbance of 2.7 at 532 nm measured on the 0.5 mm thick sample equals an absorption coefficient of 54 cm^{-1}.

the measured absorbance can be given using the absorption cross-section.

The absorbance of our 0.5 mm thick sample at this wavelength is about 2.7, which yields an absorption coefficient of $54\,\mathrm{cm}^{-1}$. The absorption cross-section values of NV centers in the visible regime range between 2.8×10^{-17} cm^{-2} and 9.5×10^{-17} cm^{-2} reported in Refs. (*51–54*). The corresponding number of NV centers in a unit volume is between 4.4×10^{18} cm^{-3} and 1.3×10^{18} cm^{-3} (corresponding to 25.2 ppm and 7.4 ppm respectively). We note that the

absorption cross-section value of $5.7 \pm 0.8 \times 10^{-17}$ cm^{-2} in reported Ref. (*52*) gives 12.4 ppm concentration that matches our value of 12 ppm determined with CW ESR measurement.

8 Rotation of ISC transition rates into the laboratory frame in high magnetic field.

In high magnetic field, the Zeeman interaction is far stronger than that of the ZFS. It is convenient to define the laboratory frame that aligns with the direction of the magnetic field: $\boldsymbol{B} = (0, 0, B)^T$. Zeeman interaction is diagonal thus giving the $|-1\rangle_{\boldsymbol{B}}$, $|0\rangle_{\boldsymbol{B}}$, $|+1\rangle_{\boldsymbol{B}}$ eigenstates separated from each by $g_e\mu_B$ where $\boldsymbol{B}$ in the subscript depicts the magnetic field oriented frame of reference. If the applied $\boldsymbol{B}$-field is oriented towards the $[111]$ symmetry axis then NV's own coordinate system and the laboratory frame would coincide thus the $\Gamma_{\pm}$, Γ_z rates shown in Fig. 1 are acting between $|m\rangle_{\boldsymbol{B}}$, where $m = -1, 0, +1$. However, if the $[111]$ oriented $\boldsymbol{B}$-field exerts its effect on one of the three remaining possible NV orientations: $[1\bar{1}\bar{1}]$, $[\bar{1}1\bar{1}]$, $[\bar{1}\bar{1}1]$ we need to rotate the inter-system crossing (ISC) rates.

The rotation matrix $R(\alpha, \beta, \gamma)$ can be described by three consecutive rotations depicted by three (α, β, γ) Euler angles:

$$\begin{pmatrix} 0 \\ 0 \\ B \end{pmatrix}_{\boldsymbol{B}} = \underbrace{R_{\hat{z}}(\alpha) R_{\hat{n}}(\beta) R_{\hat{z}}(\gamma)}_{R(\alpha,\beta,\gamma)} \begin{pmatrix} B_x \\ B_y \\ B_z \end{pmatrix}_{\text{NV}}, \tag{13}$$

where we follow the "passive" convention. According to Refs. (*55, 56*) any irreducible tensor transforming as $S = 1$ representation defined within NV's frame can be transformed into the $\boldsymbol{B}$-field's quantization axis by the following Wigner matrix,

$$D^{S=1}_{\boldsymbol{B},\text{NV}}(\alpha, \beta, \gamma) = \begin{pmatrix} \frac{1+\cos(\beta)}{2} \mathrm{e}^{-\mathrm{i}(\alpha+\gamma)} & -\frac{1}{\sqrt{2}} \sin(\beta) \mathrm{e}^{-\mathrm{i}\alpha} & \frac{1-\cos(\beta)}{2} \mathrm{e}^{-\mathrm{i}(\alpha-\gamma)} \\ \frac{1}{\sqrt{2}} \sin(\beta) \mathrm{e}^{-\mathrm{i}\gamma} & \cos(\beta) & -\frac{1}{\sqrt{2}} \sin(\beta) \mathrm{e}^{\mathrm{i}\gamma} \\ \frac{1-\cos(\beta)}{2} \mathrm{e}^{\mathrm{i}(\alpha-\gamma)} & \frac{1}{\sqrt{2}} \sin(\beta) \mathrm{e}^{\mathrm{i}\alpha} & \frac{1+\cos(\beta)}{2} \mathrm{e}^{\mathrm{i}(\alpha+\gamma)} \end{pmatrix} \tag{14}$$

that can be used to transform the spin of the three eigenstates as $|m\rangle_{\text{NV}} = \sum_{m'} \left[D^{S=1}_{\boldsymbol{B},\text{NV}}(\alpha, \beta, \gamma) \right]_{m,m'} |m\rangle'_{\boldsymbol{B}}$. Therefore, now we can transform the interaction $\hat{W}$ that

drives the non-radiative process between a singlet $|s\rangle$ and the triplet levels $\langle m|$ with involving the phonon operators $\hat{R}_m$ as $\hat{W} = \sum_m t_m^{\text{NV}} |s\rangle \langle m|_{\text{NV}} \otimes \hat{R}_m + \text{h.c.}$ to create the appropriate amount of phonons that absorb the energy gain by relaxing from the higher electronic state to the lower electronic state and receive the angular momentum difference m between the two electronic states. However, while it seems convenient but surprisingly the aforementioned Wigner transformation on t_m^{NV} transition amplitudes should be further considered in terms of the phase. The reason is the following. The phase of individual phonons depicted by $\hat{R}_m$ is random, thus the amplitudes cannot be added directly. This leads to the random phase approximation,

$$\left|t_m^{\boldsymbol{B}}\right|^2 = \sum_m \left|D_{\boldsymbol{B},\text{NV}}^{S=1}(\alpha, \beta, \gamma)_{m,m'}\right|^2 \left|t_m^{\text{NV}}\right|^2, \tag{15}$$

where one has to transform the absolute squares of the transition amplitudes, i.e., the rates with the absolute square of the Wigner matrix as shown below,

$$\left|D_{\boldsymbol{B},\text{NV}}^{S=1}(\alpha, \beta, \gamma)_{m,m'}\right|^2 = \begin{pmatrix} \cos^4(\beta/2) & \frac{1}{2}\sin^2(\beta) & \sin^4(\beta/2) \\ \frac{1}{2}\sin^2(\beta) & \cos^2(\beta) & \frac{1}{2}\sin^2(\beta) \\ \sin^4(\beta/2) & \frac{1}{2}\sin^2(\beta) & \cos^4(\beta/2) \end{pmatrix}.$$

We note that the rotated transition rates are only dependent on the polar angle β that rotates the system out from NV's [111] direction and independent on the two azimuthal angles α, γ that leave the $\boldsymbol{B}$-field's direction intact during their rotation. Therefore, it is not surprising that the transformed rates depend only on the angle between NV's and $\boldsymbol{B}$-field's direction. As mentioned in the main text the external magnetic field is oriented parallel to the symmetry axis of one NV configuration whereas the other three NV configurations exhibit $\beta = 109.5°$ angle with respect to the magnetic field direction that leads to the following transformation rules for the intersystem crossing of the three off-axis configurations for the upper branch from $|^3E\rangle$ to $|^1A_1\rangle$,

$$\begin{pmatrix} \Gamma_{\text{ISC}} \\ 0 \\ \Gamma_{\text{ISC}} \end{pmatrix}_{\text{NV}} \rightarrow \begin{pmatrix} \Gamma_+ \\ \Gamma_0 \\ \Gamma_- \end{pmatrix}_{\boldsymbol{B}} = \frac{1}{9} \begin{pmatrix} 5\Gamma_{\text{ISC}} \\ 8\Gamma_{\text{ISC}} \\ 5\Gamma_{\text{ISC}} \end{pmatrix}_{\boldsymbol{B}}, \tag{16}$$

and for the lower branch acting between $|^1E\rangle$ and $|^3A_2\rangle$,

$$\begin{pmatrix} \Gamma_\perp \\ \Gamma_z \\ \Gamma_\perp \end{pmatrix}_{\text{NV}} \to \begin{pmatrix} \Gamma'_+ \\ \Gamma'_0 \\ \Gamma'_- \end{pmatrix}_{\boldsymbol{B}} = \frac{1}{9} \begin{pmatrix} 5\Gamma_\perp + 4\Gamma_z \\ 8\Gamma_\perp + 1\Gamma_z \\ 5\Gamma_\perp + 4\Gamma_z \end{pmatrix}_{\boldsymbol{B}} . \tag{17}$$

Here, $\Gamma_z \approx 1.2\times\Gamma_\perp$ according to previous theoretical findings (*49*). Recently, the ratio between the rates was measured (*57*) as $\Gamma_z \approx 2 \times \Gamma_\perp$. On the other hand, we assume that the transition rates for $|0\rangle$ spin projections are zeroes (*28*) for the excited triplet state. The transition rate for $|0\rangle$ is at least a magnitude smaller (*58,59*) than that of $|\pm1\rangle$. The measured (*59,60*) rate for $|\pm1\rangle$ is $\Gamma_{\text{ISC}} = 2\pi \times \frac{1}{4}(\Gamma_{A_1} + 2\Gamma_{E_{1,2}}) = \frac{2\pi}{4}(16.0 + 2 \times 8.3)$ MHz $= 51.2$ MHz while the rate for $|0\rangle$ is $\Gamma^{(0)}_{\text{ISC}} = 2\pi \times (0.62 \pm 0.21)$ MHz $= 3.90 \pm 1.32$ MHz. We note that the ratio between Γ_0/Γ_+ does not change significantly due to this. Eq. (16) depicts $\Gamma_0/\Gamma_+ = 1.2$ while with additional $\Gamma^{(0)}_{\text{ISC}}$ it would be $\Gamma_0/\Gamma_+ = 1.18$. Therefore, we omit $\Gamma^{(0)}_{\text{ISC}}$ completely from our discussion for simplicity. The result is depicted in Fig. S8.

Additionally, we assume that the orbital doublets $|^1E\rangle$ and $|^3E\rangle$ are thermally averaged over their twofold orbital degrees of freedom. Thus, we treat $|^1E\rangle$ as a single quantum state effectively from which the $\Gamma_\perp$, Γ_z transition rates originate. Similarly, the upper ISC rate depends only on the spin wavefunction as the orbital degeneracy in $|^3E\rangle$ is averaged out.

As it is described in Eqs. (16) and (17) that the misoriented NV configurations exhibit a much more balanced ISC rate pattern than that of the [111] oriented NV centers. Therefore, the effect of LESR is not as significant for transitions 2 and 3 (off-axis configurations) as for transitions 1 and 4 (parallel configurations) in Fig. 2 b. For the parallel configuration, optical spin-polarization will preferentially populate $|0\rangle$ state which leads to an induced emission for transition "4" ($|0\rangle \to |-1\rangle$ transition). For the off-axis configurations, the preferential population turns towards $|\pm1\rangle$ thus induced emission is observed for transition "3" ($|1\rangle \to |0\rangle$ transition) in Fig. 2b.

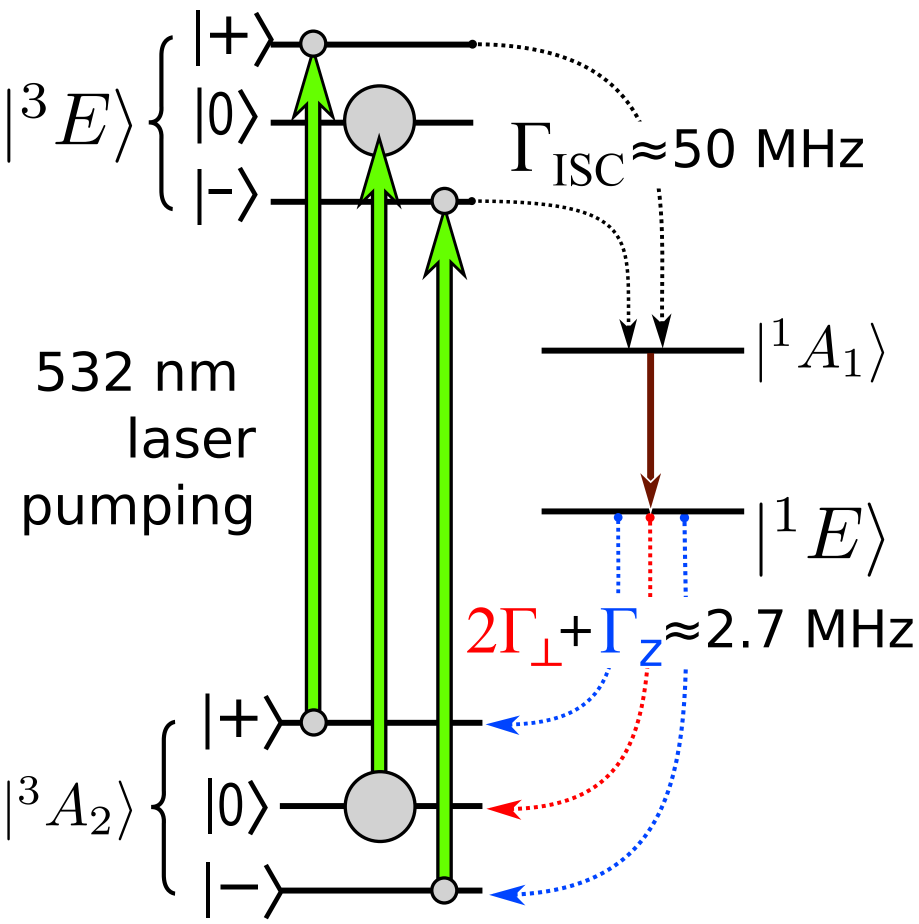

Figure S8: **Optical spin polarization cycle of NV centers**.

9 Outline and critical assessment of a diamond NV based TASER system.

The construction of a THz laser (TASER in the following) system based on diamond NV centers could closely follow a microcavity laser design. The proposed design is shown in Fig. S9.

The magnetic resonance selection rules dictate that the polarization plane of the absorbed or generated THz radiation is perpendicular to the external magnetic field. Efficient lasing was observed with such a geometry in Ref. (*21*). This is the same geometry in which we observed the THz emission in the diamond NV centers.

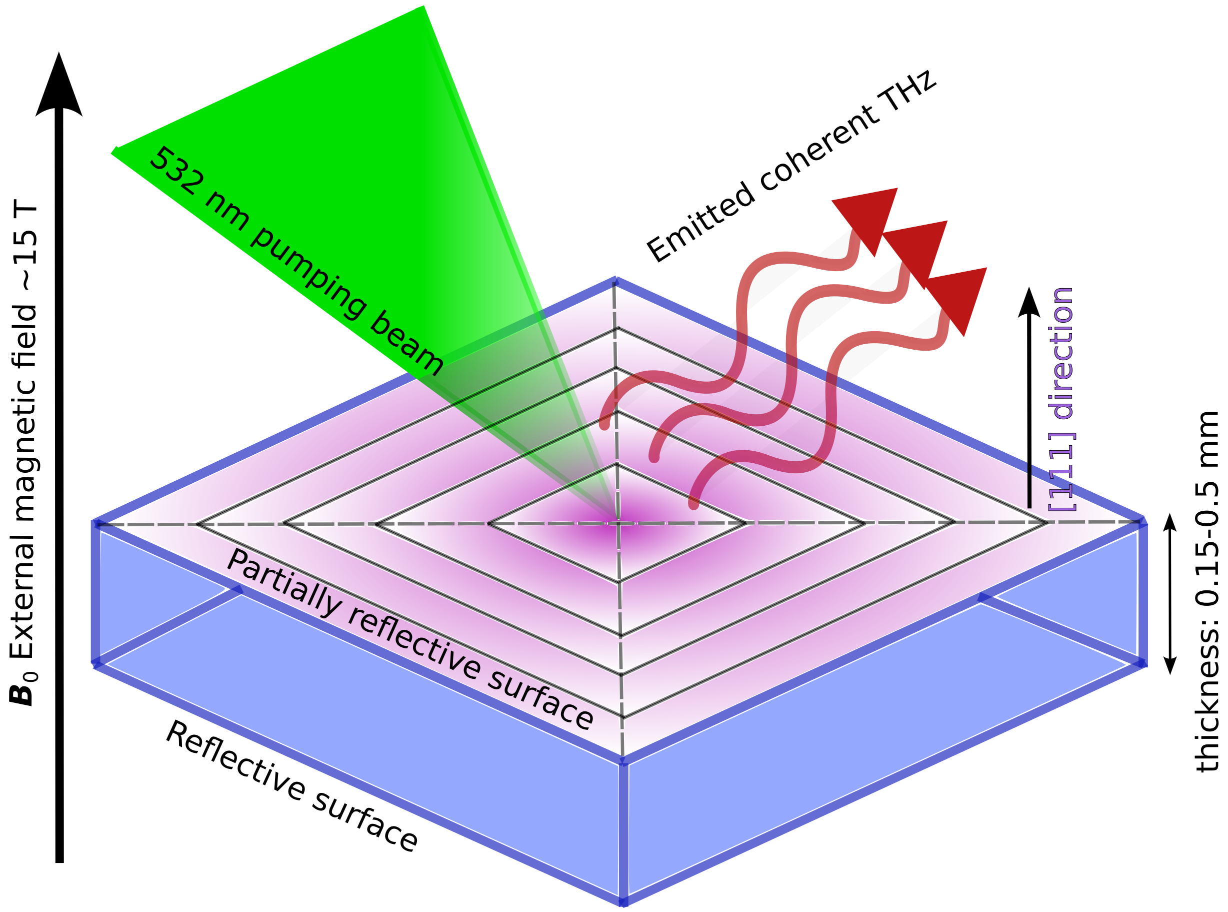

Figure S9: **Proposed construction of a 0.4 THz TASER.**

More specifically, this is a so-called Fabry-Pérot microcavity design (*61*). The working condition for the Fabry-Pérot microcavity is $\lambda = 2L/m$, where L is the thickness of the microcavity, m is a positive integer, λ is the wavelength of the generated radiation in the medium.

The proposed geometry is also optimal for the pumping efficiency, as the polarization of the incoming (pumping) light is perpendicular to the NV axis (*40*). We focus on a TASER operating at 0.4 THz, as it can be achieved with a conventional laboratory magnetic field of around 15 T. The corresponding wavelength is $\lambda(0.4\,\mathrm{THz}) = 0.314\,\mathrm{mm}$ using the index of refraction $n = 2.38$ in the THz range for diamond (*62*). This means that a microcavity, which sustains 0.4 THz TASER modes, has a thickness of $L = 0.157\,\mathrm{mm}$ or its multiples.

In fact, a thickness between 0.15-0.5 mm is ideal for a sample with the NV concentration such as in our sample due to the above described 54 cm^{-1} absorption coefficient measured at 532 nm. This means that a sample with a thickness of 0.2 mm absorbs $1/e$-th part of the exciting laser. The pumping light would not be exploited optimally for a thinner sample. For a much thicker sample, the NV centers away from the sample surface would be pumped less efficiently by the attenuated power of the pump laser.

The key factor in determining whether the TASER operation can be sustained is the cooperativity (*21*) factor: $C = 2PN \cdot T_2^*$. Here, P is the Purcell factor, N is the number of active NV centers and T_2^* is the spin dephasing factor. The latter usually describes the inverse of the observable linewidth in magnetic resonance experiments. The Purcell factor is well approximated by $P \approx 0.1Q\frac{\lambda^3}{V}$, where Q is the quality factor of the microcavity resonator, V is its volume and λ is the wavelength of the emitted radiation inside the cavity.

While obtaining absolute estimates for these values is difficult, we make a comparison with Ref. (*21*), where microwave lasing (or masing) was successfully achieved. The concentration in our sample is about 30 times larger than in Ref. (*21*) we thus estimate that the number of active NV centers is similarly larger herein. We estimate that the Purcell factor for the proposed TASER design should be similar to that in Ref (*21*). It goes essentially with the volume filling factor of the sample with respect to the resonator, which sustains the radiation. For both the maser and TASER designs, the two are around unity. Typical microwave quality factors are a few thousand which is also feasible in microcavity resonators. Therefore, we expect a similar Purcell factor for the two designs. The last remaining factor, which requires careful consideration is the dephasing rate. In electron spin resonance on solids, the dephasing rate or $1/T_2^*$ arises mainly from impurities and unresolved hyperfine couplings (*47*). This is markedly different from the case of nuclear magnetic resonance (especially for liquids) where the dominant contribution to $1/T_2^*$ is due to the magnetic field inhomogeneity. Were this also the case herein,

one would expect that the higher magnetic field results in a much higher dephasing rate (about 16 T/0.3 T$\approx$ 50 times higher). However, the dephasing effects of impurities or unresolved hyperfine couplings are inherently field *independent* (*36*), we therefore expect similar dephasing rates for the proposed TASER than in Ref. (*21*). Altogether, this consideration gives that about 30 times higher cooperativity could be achieved in our proposed design, which predicts the operation of an efficient THz laser.

REFERENCES AND NOTES

1. M. Beck, M. Klammer, S. Lang, P. Leiderer, V. V. Kabanov, G. N. Gol'tsman, J. Demsar, Energy-Gap dynamics of superconducting NbN thin films studied by time-resolved terahertz spectroscopy. *Phys. Rev. Lett.* **107**, 177007 (2011).
2. N. Kida, M. Tonouchi, THz spectroscopic evidence for a charge-density-wave formation in a charge-ordered manganite $Pr_{0.7}$ $Ca_{0.3}$ MnO_3. *Phys. B* **329–333**, 842–843 (2003).
3. K. Grishunin, T. Huisman, G. Li, E. Mishina, T. Rasing, A. V. Kimel, K. Zhang, Z. Jin, S. Cao, W. Ren, G.-H. Ma, R. V. Mikhaylovskiy, Terahertz magnon-polaritons in TmFeO(3). *ACS Photonics* **5**, 1375–1380 (2018).
4. P. Kusar, V. V. Kabanov, J. Demsar, T. Mertelj, S. Sugai, D. Mihailovic, Controlled vaporization of the superconducting condensate in cuprate superconductors by femtosecond photoexcitation. *Phys. Rev. Lett.* **101**, 227001 (2008).
5. J. Ravnik, M. Diego, Y. Gerasimenko, Y. Vaskivskyi, I. Vaskivskyi, T. Mertelj, J. Vodeb, D. Mihailovic, A time-domain phase diagram of metastable states in a charge ordered quantum material. *Nat. Comm.* **12**, 2323 (2021).
6. S. S. Dhillon, M. S. Vitiello, E. H. Linfield, A. G. Davies, M. C. Hoffmann, J. Booske, C. Paoloni, M. Gensch, P. Weightman, G. P. Williams, E. Castro-Camus, D. R. S. Cumming, F. Simoens, I. Escorcia-Carranza, J. Grant, S. Lucyszyn, M. Kuwata-Gonokami, K. Konishi, M. Koch, C. A. Schmuttenmaer, T. L. Cocker, R. Huber, A. G. Markelz, Z. D. Taylor, V. P. Wallace, J. A. Zeitler, J. Sibik, T. M. Korter, B. Ellison, S. Rea, P. Goldsmith, K. B. Cooper, R. Appleby, D. Pardo, P. G. Huggard, V. Krozer, H. Shams, M. Fice, C. Renaud, A. Seeds, A. Stoehr, M. Naftaly, N. Ridler, R. Clarke, J. E. Cunningham, M. B. Johnston, The 2017 terahertz science and technology roadmap. *J. Phys. D Appl. Phys.* **50**, 043001 (2017).
7. D. H. Auston, K. P. Cheung, J. A. Valdmanis, D. A. Kleinman, Cherenkov radiation from femtosecond optical pulses in electro-optic media. *Phys. Rev. Lett.* **53**, 1555–1558 (1984).
8. D. W. Faries, K. A. Gehring, P. L. Richards, Y. R. Shen, Tunable far-infrared radiation generated from the difference frequency between two ruby lasers. *Phys. Rev.* **108**, 363–365 (1969).
9. U. Welp, K. Kadowaki, R. Kleiner, Superconducting emitters of THz radiation. *Nat. Photonics* **7**, 702–710 (2013).
10. N. Orihashi, S. Suzuki, M. Asada, One THz harmonic oscillation of resonant tunneling diodes. *Appl. Phys. Lett.* **87**, 233501 (2005).

11. J. H. Booske, R. J. Dobbs, C. D. Joye, C. L. Kory, G. R. Neil, G.-S. Park, J. Park, R. J. Temkin, Vacuum electronic high power terahertz sources. *IEEE Trans. Terahertz Sci. Technol.* **1**, 54–75 (2011).

12. R. Kohler, A. Tredicucci, F. Beltram, H. Beere, E. Linfield, A. Davies, D. Ritchie, R. Iotti, F. Rossi, Terahertz semiconductor-heterostructure laser. *Nature* **417**, 156–159 (2002).

13. G. Williams, Filling the THz gap—High power sources and applications. *Rep. Prog. Phys.* **69**, 301–326 (2006).

14. A. Fisher, Y. Park, M. Lenz, A. Ody, R. Agustsson, T. Hodgetts, A. Murokh, P. Musumeci, Single-pass high-efficiency terahertz free-electron laser. *Nat. Photonics* **16**, 441–447 (2022).

15. T. H. Maiman, Stimulated optical radiation in ruby. *Nature* **187**, 493–494 (1960).

16. A. Gruber, A. Dräbenstedt, C. Tietz, L. Fleury, J. Wrachtrup, C. Borczyskowski, Scanning confocal optical microscopy and magnetic resonance on single defect centers. *Science* **276**, 2012–2014 (1997).

17. N. B. Manson, J. P. Harrison, M. J. Sellars, Nitrogen-vacancy center in diamond: Model of the electronic structure and associated dynamics. *Phys. Rev. B* **74**, 104303 (2006).

18. A. P. Nizovtsev, S. Y. Kilin, F. Jelezko, T. Gaebal, I. Popa, A. Gruber, J. Wrachtrup, A quantum computer based on nv centers in diamond: Optically detected nutations of single electron and nuclear spins. *Opt. Spectrosc.* **99**, 233–244 (2005).

19. L. M. Pham, D. L. Sage, P. L. Stanwix, T. K. Yeung, D. Glenn, A. Trifonov, P. Cappellaro, P. R. Hemmer, M. D. Lukin, H. Park, A. Yacoby, R. L. Walsworth, Magnetic field imaging with nitrogen-vacancy ensembles. *New J. Phys.* **13**, 045021 (2011).

20. A. Jarmola, V. M. Acosta, K. Jensen, S. Chemerisov, D. Budker, Temperature- and magnetic-field-dependent longitudinal spin relaxation in nitrogen-vacancy ensembles in diamond. *Phys. Rev. Lett.* **108**, 197601 (2012).

21. J. D. Breeze, E. Salvadori, J. Sathian, N. M. Alford, C. W. M. Kay, Continuous-wave room-temperature diamond maser. *Nature* **555**, 493–496 (2018).

22. A. Sherman, O. Zgadzai, B. Koren, I. Peretz, E. Laster, A. Blank, Diamond-based microwave quantum amplifier. *Sci. Adv.* **8**, 6527 (2022).

23. H. Kraus, V. A. Soltamov, D. Riedel, S. Vaeth, F. Fuchs, A. Sperlich, P. G. Baranov, V. Dyakonov, G. V. Astakhov, Room-temperature quantum microwave emitters based on spin defects in silicon carbide. *Nat. Phys.* **10**, 157–162 (2014).

24. G. Davies, M. F. Hamer, Optical studies of the 1.945 eV vibronic band in diamond. *Proc. R. Soc. Lond. A* **348**, 285–298 (1976).

25. J. H. N. Loubser, J. A. van Wyk, Electron spin resonance in the study of diamond. *Rep. Prog. Phys.* **41**, 1201–1248 (1978).
26. Y. Mita, Change of absorption spectra in type-ib diamond with heavy neutron irradiation. *Phys. Rev. B* **53**, 11360–11364 (1996).
27. J. A. Larsson, P. Delaney, Electronic structure of the nitrogen-vacancy center in diamond from first-principles theory. *Phys. Rev. B* **77**, 165201 (2008).
28. G. Thiering, A. Gali, Ab initio calculation of spin-orbit coupling for an NV center in diamond exhibiting dynamic Jahn-Teller effect. *Phys. Rev. B* **96**, 081115(R) (2017).
29. J. Harrison, M. Sellars, N. Manson, Optical spin polarisation of the N-V centre in diamond. *J. Lumin.* **107**, 245–248 (2004).
30. B. Náfrádi, R. Gaál, T. Fehér, L. Forró, Microwave frequency modulation in continuous-wave far-infrared ESR utilizing a quasi-optical reflection bridge. *J. Magn. Reson.* **192**, 265–268 (2008).
31. B. Náfrádi, R. Gaál, A. Sienkiewicz, T. Fehér, L. Forró, Continuous-wave far-infrared ESR spectrometer for high-pressure measurements. *J. Magn. Reson.* **195**, 206–210 (2008).
32. S. Kollarics, F. Simon, A. Bojtor, K. Koltai, G. Klujber, M. Szieberth, B. G. Márkus, D. Beke, K. Kamarás, A. Gali, D. Amirari, R. Berry, S. Boucher, D. Gavryushkin, G. Jeschke, J. Cleveland, S. Takahashi, P. Szirmai, L. Forró, E. Emmanouilidou, R. Singh, K. Holczer, Ultrahigh nitrogen-vacancy center concentration in diamond. *Carbon* **188**, 393–400 (2022).
33. C. P. Slichter, *Principles of Magnetic Resonance* (Spinger-Verlag, ed. 3, 1996).
34. S. Felton, A. M. Edmonds, M. E. Newton, P. M. Martineau, D. Fisher, D. J. Twitchen, J. M. Baker, Hyperfine interaction in the ground state of the negatively charged nitrogen vacancy center in diamond. *Phys. Rev. B* **79**, 075203 (2009).
35. C. P. Poole, *Electron Spin Resonance* (John Wiley & Sons, ed. 2, 1983).
36. A. Abragam, *Principles of Nuclear Magnetism* (Oxford Univ. Press, 1961), vol. **29**, pp. 860–861.
37. M.-K. Lee, M. Segal, Z. G. Soos, J. Shinar, M. A. Baldo, Yield of singlet excitons in organic light-emitting devices: A double modulation photoluminescence-detected magnetic resonance study. *Phys. Rev. Lett.* **94**, 137403 (2005).
38. V. Stepanov, F. H. Cho, C. Abeywardana, S. Takahashi, High-frequency and high-field optically detected magnetic resonance of nitrogen-vacancy centers in diamond. *Appl. Phys. Lett.* **106**, 063111 (2015).

39. R. Epstein, F. Mendoza, Y. Kato, D. Awschalom, Anisotropic interactions of a single spin and dark-spin spectroscopy in diamond. *Nat. Phys.* **1**, 94–98 (2005).
40. T. P. M. Alegre, C. Santori, G. Medeiros-Ribeiro, R. G. Beausoleil, Polarization-selective excitation of nitrogen vacancy centers in diamond. *Phys. Rev. B* **76**, 165205 (2007).
41. L. Rondin, J.-P. Tetienne, T. Hingant, J.-F. Roch, P. Maletinsky, V. Jacques, Magnetometry with nitrogen-vacancy defects in diamond. *Rep. Prog. Phys.* **77**, 056503 (2014).
42. T. D. Ladd, F. Jelezko, R. Laflamme, Y. Nakamura, C. Monroe, J. L. O'Brien, Quantum computers. *Nature* **464**, 45–53 (2010).
43. B. G. Márkus, M. Gmitra, B. Dóra, G. Csősz, T. Fehér, P. Szirmai, B. Náfrádi, V. Zólyomi, L. Forró, J. Fabian, F. Simon, Ultralong 100 ns spin relaxation time in graphite at room temperature. *Nat. Commun.* **14**, 2831 (2023).
44. B. Fortman, L. Mugica-Sanchez, N. Tischler, C. Selco, Y. Hang, K. Holczer, S. Takahashi, Electron–electron double resonance detected nmr spectroscopy using ensemble nv centers at 230 ghz and 8.3 t. *J. Appl. Phys.* **130**, 083901 (2021).
45. S. Hallstein, J. D. Berger, M. Hilpert, H. C. Schneider, W. W. Rühle, F. Jahnke, S. W. Koch, H. M. Gibbs, G. Khitrova, M. Oestreich, Manifestation of coherent spin precession in stimulated semiconductor emission dynamics. *Phys. Rev. B* **56**, R7076–R7079 (1997).
46. M. Lindemann, G. Xu, T. Pusch, R. Michalzik, M. R. Hofmann, I. Žutić, N. C. Gerhardt, Ultrafast spin-lasers. *Nature* **568**, 212–215 (2019).
47. A. Portis, Spectral diffusion in magnetic resonance. *Phys. Rev.* **104**, 584–588(1956).
48. P. A. Lane, L. S. Swanson, Q.-X. Ni, J. Shinar, J. P. Engel, T. J. Barton, L. Jones, Dynamics of photoexcited states in C_{60}: An optically detected magnetic resonance, ESR, and light-induced ESR study. *Phys. Rev. Lett.* **68**, 887–890 (1992).
49. G. Thiering, A. Gali, Theory of the optical spin-polarization loop of the nitrogen-vacancy center in diamond. *Phys. Rev. B* **98**, 085207 (2018).
50. J. Goree, Double lock-in detection for recovering weak coherent radio frequency signals. *Rev. Sci. Instrum.* **56**, 1662–1664 (1985).
51. S. D. Subedi, V. V. Fedorov, J. Peppers, D. V. Martyshkin, S. B. Mirov, L. Shao, M. Loncar, Laser spectroscopic characterization of negatively charged nitrogen-vacancy (NV-) centers in diamond. *Opt. Mater. Express* **9**, 2076–2087 (2019).

52. A. Savvin, A. Dormidonov, E. Smetanina, V. Mitrokhin, E. Lipatov, D. Genin, S. Potanin, A. Yelisseyev, V. Vins, Nv–diamond laser. *Nat. Comm.* **12**, 7118 (2021).
53. T.-L. Wee, Y.-K. Tzeng, C.-C. Han, H.-C. Chang, W. Fann, J.-H. Hsu, K.-M. Chen, Y.-C. Yu, Two-photon excited fluorescence of nitrogen-vacancy centers in proton-irradiated type ib diamond. *J. Phys. Chem. A* **111**, 9379–9386 (2007).
54. R. Chapman, T. Plakhotnik, Quantitative luminescence microscopy on nitrogen-vacancy centres in diamond: Saturation effects under pulsed excitation. *Chem. Phys. Lett.* **507**, 190–194 (2011).
55. P. P. Man, Wigner active and passive rotation matrices applied to NMR tensor. *Concepts Magn. Reson.* **45A**, e21385 (2016).
56. B. C. Rose, G. Thiering, A. M. Tyryshkin, A. M. Edmonds, M. L. Markham, A. Gali, S. A. Lyon, N. P. de Leon, Strongly anisotropic spin relaxation in the neutral silicon vacancy center in diamond. *Phys. Rev. B* **98**, 235140 (2018).
57. A. Reiserer, N. Kalb, M. S. Blok, K. J. van Bemmelen, T. H. Taminiau, R. Hanson, D. J. Twitchen, M. Markham, Robust quantum-network memory using decoherence-protected subspaces of nuclear spins. *Phys. Rev. X* **6**, 021040 (2016).
58. J.-P. Tetienne, L. Rondin, P. Spinicelli, M. Chipaux, T. Debuisschert, J.-F. Roch, V. Jacques, Magnetic-field-dependent photodynamics of single NV defects in diamond: An application to qualitative all-optical magnetic imaging. *New J. Phys.* **14**, 103033 (2012).
59. M. L. Goldman, A. Sipahigil, M. W. Doherty, N. Y. Yao, S. D. Bennett, M. Markham, D. J. Twitchen, N. B. Manson, A. Kubanek, M. D. Lukin, Phonon-induced population dynamics and intersystem crossing in nitrogen-vacancy centers. *Phys. Rev. Lett.* **114**, 145502 (2015).
60. M. L. Goldman, M. W. Doherty, A. Sipahigil, N. Y. Yao, S. D. Bennett, N. B. Manson, A. Kubanek, M. D. Lukin, State-selective intersystem crossing in nitrogen-vacancy centers. *Phys. Rev. B* **91**, 165201 (2015).
61. N. Liang, J. Yan, T. Zhai, Hybrid microcavity lasers: Principle, design, and practical application. *Laser Photonics Rev.* **17**, 2300343 (2023).
62. V. Rogalin, I. Kaplunov, G. Kropotov, Optical materials for the THz range. *Opt. Spectrosc.* **125**, 1053–1064 (2018).
